# Synthetic Star Nanoengineered Antimicrobial Polymers as Antibiofilm Agents: Bacterial Membrane Disruption and Cell Aggregation

**DOI:** 10.1021/acs.biomac.3c00150

**Published:** 2023-06-10

**Authors:** Sophie Laroque, Ramón Garcia Maset, Alexia Hapeshi, Fannie Burgevin, Katherine E. S. Locock, Sébastien Perrier

**Affiliations:** † Department of Chemistry, University of Warwick, Gibbet Hill Road, Coventry CV4 7AL, U.K.; ‡ Warwick Medical School, University of Warwick, Coventry CV4 7AL, U.K.; § CSIRO Manufacturing, Clayton, Victoria 3168, Australia; ∥ Faculty of Pharmacy and Pharmaceutical Sciences, Monash University, 381 Royal Parade, Parkville, Victoria 3052, Australia

## Abstract

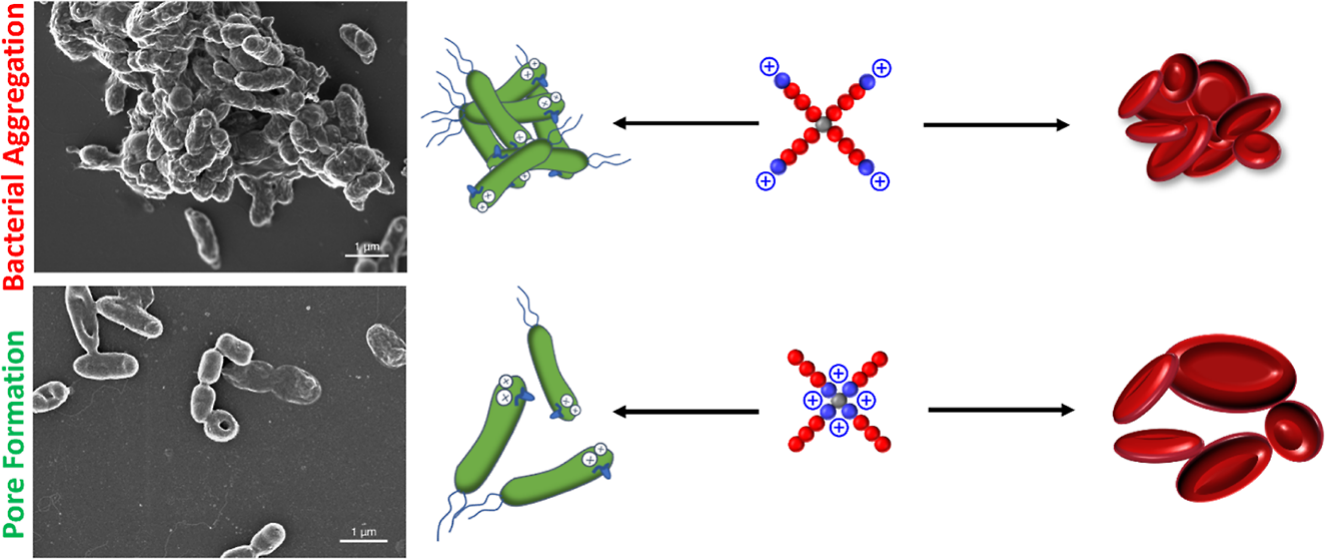

Antimicrobial resistance has become a worldwide issue, with multiresistant bacterial strains emerging at an alarming rate. Multivalent antimicrobial polymer architectures such as bottle brush or star polymers have shown great potential, as they could lead to enhanced binding and interaction with the bacterial cell membrane. In this study, a library of amphiphilic star copolymers and their linear copolymer equivalents, based on acrylamide monomers, were synthesized via RAFT polymerization. Their monomer distribution and molecular weight were varied. Subsequently, their antimicrobial activity toward a Gram-negative bacterium (*Pseudomonas aeruginosa* PA14) and a Gram-positive bacterium (*Staphylococcus aureus* USA300) and their hemocompatibility were investigated. The statistical star copolymer, S-SP25, showed an improved antimicrobial activity compared to its linear equivalent against*P. aeruginosa*PA14. The star architecture enhanced its antimicrobial activity, causing bacterial cell aggregation, as revealed via electron microscopy. However, it also induced increased red blood cell aggregation compared to its linear equivalents. Changing/shifting the position of the cationic block to the core of the structure prevents the cell aggregation effect while maintaining a potent antimicrobial activity for the smallest star copolymer. Finally, this compound showed antibiofilm properties against a robust *in vitro* biofilm model.

## 1 Introduction

The world has now entered the post-antibiotic era,^1^ in which antimicrobial resistance (AMR) has become one of the main causes of death around the globe, as 4.95 million deaths were attributed to drug-resistant infections in 2019.^2^ In a follow-up study, close to a million deaths were attributed to AMR in 2019 in Europe alone.^3^ This is expected to increase to 10 million per year by 2050 if no action is taken.^4^ A key issue is the extensive and often inappropriate use of antibiotics in clinical settings and agriculture.^5^ This problem is further aggravated by the lack of interest of the private sector in developing novel antimicrobial therapies as it is a costly and lengthy process.^6^ Additionally, the COVID-19 pandemic has further exacerbated the AMR crisis due to the increased consumption of antibiotics and biocides.^7^ Therefore, alternative treatment options to antibiotics are urgently needed.

Antimicrobial peptides (AMPs),^8^ short amphiphilic peptides with hydrophobic and cationic moieties displaying a broad spectrum of antimicrobial activities, are a promising candidate class.^9^ AMPs predominantly target the bacterial cell membrane through electrostatic interaction of the cationic amino acids with the negatively charged moieties of the membrane. This is followed by the insertion of the hydrophobic peptide units into the membrane causing disintegration and leading to cell death.^10^ Furthermore, AMPs have been shown to translocate into the bacterial cell and interact with intracellular targets such as DNA/RNA and protein synthesis.^11^ Killing of bacteria can occur synergistically with several AMPs “working together” and targeting the membrane as well as intercellular processes in a multihit mechanism.^12^ Furthermore, the antimicrobial activity of AMPs shows a sharp increase within a narrow dose range compared to antibiotics, meaning that the dose range under which resistance can be developed is very narrow.^13^

However, there are many limitations to the clinical development of AMPS, such as poor pharmacokinetic stability, degradability by enzymes, oral toxicity, laborious multistep synthesis procedures, and high production costs on industrial scales.^14^ There is great interest in overcoming these limitations, with one possibility being the synthesis of synthetic nanoengineered antimicrobial polymers (SNAPs),^15^ due to their lower production costs and higher pharmacokinetic stability.^16^ Furthermore, through advances in controlled radical polymerization over the past few decades,^17^ the ability to design and synthesize well-defined and easily tunable polymer structures has greatly enhanced their application in drug delivery and^18^ gene delivery^19^ and as antimicrobial agents.^20^ In this respect, the term nanoengineered as used in the definition of the SNAP system relates to the ability to control the microstructure of the polymers, in terms of polymeric architecture (e.g., block copolymers, star copolymers, etc.).

SNAPs aim to mimic the mode of action of AMPs by imitating their essential structural properties through the introduction of cationic and hydrophobic units.^15^ Generally, a higher ratio of cationic units results in a higher selectivity toward the negatively charged bacterial membrane, while apolarity increases the antimicrobial activity but also cytotoxicity.^21^ The balance between hydrophobic and cationic units is, therefore, the most investigated parameter in order to reach an optimal compromise between high antimicrobial activity and cytotoxicity.^22^ Furthermore, our group has determined that *N*-isopropylacrylamide (NIPAm), a hydrophilic acrylamide monomer with an apolar isopropyl side chain, yields amphiphilic copolymers with good antimicrobial activity and low cytotoxicity when copolymerized with cationic comonomers.^23−25^ This demonstrates that an isopropyl group provides enough hydrophobic character to the molecule for obtaining copolymers with antimicrobial activity, reminiscent of the role of the amino acid leucine in AMPs. The balance between activity and cytotoxicity can be greatly affected by the distribution of cationic and apolar units along the polymer chains.^26^ Indeed, it was found in a previous study conducted by our group that a block copolymer showed superior antimicrobial activity as well as lower toxicity compared to its statistical copolymer counterpart.^25^ Furthermore, in a recent study conducted by Garcia Maset et al., it was found that a triblock with a cationic center and apolar outer blocks showed great promise in binding and disrupting the inner and outer bacterial membranes of *Pseudomonas aeruginosa*.^23^

Polymer architecture is another crucial parameter modulating the antimicrobial activity and the interaction with bacterial membranes.^27,28^ The difference of higher-order polymer architectures compared to linear polymers is their multivalency, which is ubiquitously found in nature.^29^ The multivalency allows binding to multiple ligands from one entity to multiple receptors on another, which can lead to enhanced binding and interactions compared to monovalent systems, also referred to as avidity.^30^ Therefore, higher-order architectures to enhance the interaction of polymers with bacterial membranes could be obtained by designing a structure where multiple polymer chains are linked together in a star-like architecture.

Such star polymers (nanostructures with a branched architecture consisting of at least three linear chains bound to a central core and forming three-dimensional globular structures) can be easily obtained using modern polymer synthesis techniques.^31^ A number of studies investigating the use of star polymers as antimicrobial agents have reported the great potential of these architectures.^32−38^ However, most studies to date have focused on statistical star copolymers, with very few examples of stars with block copolymer arms.^36^ Our group has shown that triblock copolymers ABA with a cationic center block (B) and two outer segments (A) functionalized with apolar pendant groups have excellent antibacterial activity, which is attributed to the cationic block interacting with the bacterial membrane and the apolar groups inserting and ultimately disrupting the lipid bilayer. These findings, therefore, suggest that a star structure AnB with “arms” functionalized with apolar groups (A) and a cationic core (B) could also be highly effective in disrupting bacterial membranes.^23^

These findings, therefore, suggest that a star structure A_*n*_B with apolar “arms” (A) and a cationic core (B) could also be highly effective in disrupting bacterial membranes. In addition, there is, to date, no systematic study on the structure–property relationship of star polymer structures on antimicrobial activity, including direct comparison of the influence of statistical versus diblock structures in star copolymers.

To explore this hypothesis, we synthesized a small library of star SNAPs (s-SNAPs) based on four-armed star polymers with statistical- and block-copolymer arms. In addition to block segmentation, we varied the molecular weight by changing the length of the arms and investigated the impact of changing the position of the cationic segment from core to arms. The activity of the structures as potential antimicrobial agents toward Gram-negative and Gram-positive bacteria and their toxicity toward mammalian red blood cells (RBCs) (hemolytic activity and hemagglutination) were investigated and compared to equivalent linear chains.

## 2 Experimental Section

### 2.1 Materials

4,4′-Azobis(4-cyanovaleric acid) (ACVA), acryloyl chloride, bis(*tert*-butoxycarbonyl)-2-methyl-2-thiopseudourea, Boc-anhydride (Fluka), chloroform (CHCl_3_), dimethyl sulfoxide-*d*_6_ (DMSO, 99.5%), diethyl ether (≥99.9%, inhibitor-free), dichloromethane (DCM), ethanol, ethyl acetate (EtOAc), ethylenediamine (99%), hexane, magnesium sulfate (MgSO_4_), Müller–Hinton Broth type II (MHB cationic adjusted), NIPAM (97%), phosphate buffered saline (PBS) tablets, triethylamine (NEt_3_), trifluoro acetic acid (TFA), Triton-X, 1,4-dioxane (≥99), and concanavalin A from *Canavalia ensiformis* (Jack bean) were purchased from Sigma-Aldrich.

Corning Costar flat bottom cell culture plates (bottom: flat, clear, lid: with lid, polystyrene, no. of wells: 96, sterile, surface treatment: tissue-culture treated), defibrinated sheep blood, hexamethyldisilazane (HDMS) (electronic grade, 99+%), poly-d-lysine, Thermo Scientific 96-well round (U) bottom plate, sodium chloride, and Suprasil quartz cuvettes were purchased from Fisher Scientific.

Corning Costar TC-treated multiple well plates (24-well plates) were purchased from Merk. A round coverslip of 12 mm diameter (631-1577P) was purchased from VWR International Ltd (UK).

2′-Azobis[2-(2-imidazolin-2-yl)propane]dihydrochloride (VA-044) was purchased from Wako. Pre-wetted RC tubings 1 kD were purchased from Spectrumlabs. Glutaraldehyde solution 25% for electron microscopy was purchased from PanReac AppliChem. 2-((Butylthio)-carbonothioyl) thio propanoic acid (PABTC), 4-arm PABTC, and *N*-*t*-butoxycarbonyl-1,2-diaminoethane (BocAEAM) were synthesized and purified according to the reported literature.^25^ The bacterial isolates used (*P. aeruginosa* ATCC 15442, *Staphylococcus aureus* USA300, and *P. aeruginosa* PA14) were obtained from the library of Freya Harrison’s Laboratory, School of Life Sciences, University of Warwick.

### 2.2 Monomer Synthesis (BocAEAm)

#### 2.2.1 Synthesis of *N*-*t*-Butoxycarbonyl-1,2-diaminoethane

A solution of ethylenediamine (26.71 mL, 400 mmol, 1 equiv.) in 400 mL of DCM was added to a 1 L round bottom flask fitted with a pressure equalizing dropping funnel. After the solution was cooled to 0 °C with an ice-bath, a solution of di-*tert*-butyl dicarbonate (8.73 g, 40 mmol, 0.1 equiv) in 200 mL DCM was added dropwise over 2 h under stirring. The mixture was then allowed to warm to room temperature and left stirring overnight. The solvent was removed by rotary evaporation, and 100 mL of water was added to the residue. The white precipitate was removed by filtration, and the filtrate was saturated with sodium chloride and extracted with ethyl acetate (3 × 60 mL). The combined organic phases were dried over sodium sulfate and filtered, and then solvent was removed by rotary evaporation to yield a pale oil identified as *N*-*t*-butoxycarbonyl-1,2-diaminoethane (5.3 g, 32 mmol, 82% yield).

^1^H NMR (CDCl_3_): δ 5.1 (s, 1H, CH_2_–N**H**); 3.14 (m, 2H, C**H**_**2**_–NH), 2.78 (m, 2H, C**H**_**2**_–NH_2_), 1.37 (s, 9H, C–(C**H**_**3**_)_3_) ppm.

#### 2.2.2 Synthesis of *N*-*t*-Butoxycarbonyl-*N*′-acryloyl-1,2-diaminoethane

*N*-*t*-butoxycarbonyl-1,2-diaminoethane (5.3 g, 32 mmol, 1 equiv.) and triethylamine (3.32 mL, 20 mmol, 0.7 equiv.) were dissolved in 40 mL of chloroform in a 100 mL round bottom flask fitted with a pressure equalizing dropping funnel and cooled to 0 °C with an ice-bath while stirring. Acryloyl chloride (3.05 mL, 40 mmol, 1.2 equiv.) was dissolved with 60 mL chloroform and added dropwise over one and a half hours while stirring. After addition, the mixture was allowed to warm to room temperature and left stirring for 1 h. The solvent was removed under reduced pressure and dissolved in a minimum amount of chloroform. The solution was washed with 40 mL water, which was then extracted with chloroform (3 × 60 mL). After drying over sodium sulfate and filtration, the solvent was removed by rotary evaporation to yield a white powder identified as *N*-*t*-butoxycarbonyl-*N*′-acryloyl-1,2-diaminoethane (6.2 g, 28 mmol, 88% yield).

^1^H NMR (CDCl_3_): δ 6.28 (d, 1H, CH=C**H**_**2**_), 6.11 (q, 1H, C**H**=CH_2_), 5.66 (d, 1H, CH=C**H**_**2**_), 5.1 (s, 1H, CH_2_–N**H**); 3.46 (m, 2H, NH–C**H**_**2**_–CH_2_), 3.35 (m, 2H, NH–CH_2_–C**H**_**2**_), 1.46 (s, 9H, C–(C**H**_**3**_)_3_) ppm.

^13^C NMR (CDCl_3_): δ 132 (**C**H=CH_2_) 127 (CH=**C**H_2_), 79 (O–**C**–(−CH_3_)_3_), 42 (-NH–**C**H_2_–CH_2_−), 40 (-NH–CH_2_–**C**H_2_−), 26 (O–C–(−**C**H_3_)_3_) ppm.

### 2.3 CTA Synthesis (4 Arm PABTC)

#### 2.3.1 Synthesis of Precursor (**1**)

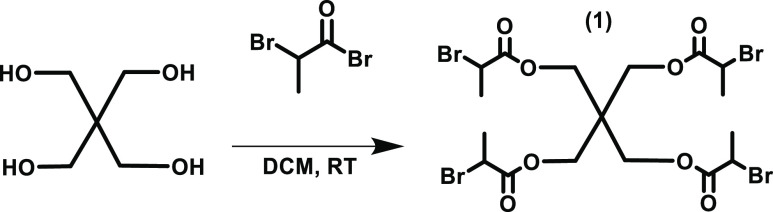



Pentaerythritol (1.183 g, 8.7 mmol, 1 equiv) and triethylamine (7.264 mL, 52 mmol, 6 equiv.) were dissolved in 100 mL DCM in a round bottom flask fitted with a pressure equalizing dropping funnel and cooled to 0 °C with an ice-bath while stirring. 2-Bromopropionyl bromide (7.281 mL, 69 mmol, 8 equiv.) was dissolved in 20 mL DCM and added dropwise over 1 h while stirring and left to stir overnight at room temperature. The dark orange-brown liquid was then filtered, and the filtrate was washed with aqueous 1 M HCl solution (3 × 80 mL), aqueous 1 M NaOH solution (3 × 80 mL), water (3 × 80 mL), and brine (3 × 80 mL). The organic fraction was dried over MgSO_4_and filtered, and the solvent was removed under reduced pressure to yield a brown solid (5.4 g, 79 mmol, 92% yield).

^1^H NMR (CDCl_3_): δ 4.36 (m, 8H, −O–C**H**_**2**_–C−), 4.23 (m, 4H, CH_3_–C**H**–Br), 1.82 (d, 12H, C**H**_**3**_–CH–Br) ppm.

#### 2.3.2 Synthesis of a Four-Armed Chain Transfer Agent (**2**)

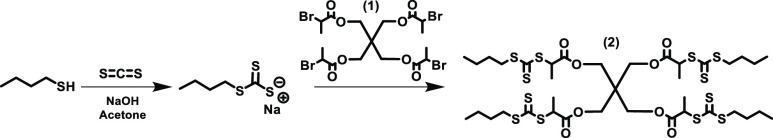



Sodium hydroxide (1.28 g, 32 mmol, 1.1 equiv.) was dissolved in 10 mL deionized water (50% w/w). Butanethiol (3.12 mL, 29 mmol, 1 equiv.) was dissolved in 20 mL acetone in a round bottom flask. The sodium hydroxide solution was slowly added, and the mixture was left to stir for 30 min. Carbon disulfide (1.79 mL, 30 mmol, 1 equiv.) was slowly added dropwise over 30 min, and then the solution was cooled down to 0 °C with an ice-bath. The precursor (1) (5.4 g, 8 mmol, 0.275 equiv.) was dissolved in 20 mL acetone, and the mixture was added dropwise over 1 h while stirring and left to stir overnight at room temperature. Dichloromethane was added to the solution, and the water was removed through a separating funnel. The organic phase was dried over magnesium sulphate, and the solvent was removed under reduced pressure to yield a yellow oil (2). The product was purified by column chromatography (hexane/ethyl acetate, gradient: 100% hexane to 80% ethyl acetate) to yield a yellow oil as the final product (4.2 g, 41 mmol, 41% yield). The IR spectrum shows no residual alcohol at 3000 cm^–1^ and an ester bond at 1736 cm^–1^ (Figure S4).

^1^H NMR (CDCl_3_): δ 4.82 (q, 1H, S–C**H**–CH_3_), 4.19–3.95 (m, 2H, −O–C**H**_**2**_–C−), 3.46–3.24 (m, 2H, S–C**H**_**2**_–CH_2_), 1.74–1.61 (m, 2H, S–CH_2_–C**H**_**2**_), 1.58 (d, 3H C**H**_**3**_–CH–S), 1.51–1.34 (m, 2H, −CH_2_–C**H**_**2**_–CH_3_), 0.93 (t, 3H, −CH_2_–CH_2_–C**H**_**3**_) ppm.

^13^C NMR (CDCL_3_): δ 222 ((S−)_2_**C**=S), 171 (O–**C**=O), 63 (C–**C**H_2_–O), 47 (CH_3_–**C**H–S), 43 (**C**(−CH_2_)_4_), 37 (S–**C**H_2_–CH_2_–CH_2_–CH_3_), 30 (S–CH_2_–**C**H_2_–CH_2_–CH_3_), 22 (S–CH_2_–CH_2_–**C**H_2_–CH_3_), 17 (**C**H_3_–CH–S), 14 (S–CH_2_–CH_2_–CH_2_–**C**H_3_) ppm.

### 2.4 General Procedure of RAFT Polymerization of Four-Armed Star Copolymers and Linear Copolymers

The monomer(s) NIPAm and/or Boc-amino ethyl acrylamide (BocAEAm), the four-armed PABTC chain transfer agent (CTA), 2,2′-azobis[2-(2-imidazolin-2-yl)propane] dihydrochloride (VA-044), and dioxane/water mixture (4:1) were added to a 5 mL glass vial equipped with a rubber septum to obtain a solution with a total concentration of 2 mol L^–1^. The solution was degassed with nitrogen for 15 min, and the reaction was heated in an oil bath to 46 °C. After 6 h had passed, the vial was removed from the oil bath and the reaction was quenched by exposure to oxygen. For kinetic reactions, the samples were taken with a syringe under nitrogen pressure at selected time points over the course of the reaction. For linear copolymers, PABTC was used as the CTA.

All four-armed linear copolymers and four-armed star diblock copolymers were synthesized with 4,4′-azobis(4-cyanovaleric acid) (ACVA) as the initiator and dioxane as the solvent. For the chain extensions, the first block was redissolved in dioxane, and the second monomer and ACVA initiator were added to make up a solution with a total concentration of 0.5 mol^–1^ mL.

### 2.5 General Procedure for the Deprotection of Boc-Protected Polymers

The polymers were dissolved in 1.5 mL of DCM and 1.5 mL of TFA, heated to 40 °C, and left to stir for 2 h. The TFA was removed by precipitation in diethyl ether. Subsequently, the polymers were dissolved in 10 mL of deionized water and dialyzed against an aqueous solution of sodium chloride with three water changes every 3 h, followed by a dialysis against water with water changes three times every 3 h. Finally, the polymers were freeze-dried.

### 2.6 Size Exclusion Chromatography

An Agilent Infinity II MDS instrument equipped with differential refractive index, viscometry, dual angle light scatter, and variable wavelength UV detectors was used. The system was equipped with 2× PLgel Mixed D columns (300 × 7.5 mm) and a PLgel 5 μm guard column. The eluent was DMF with 5 mmol NH_4_BF_4_ additive. Samples were run at 1 mL min^–1^ at 50 °C. Poly(methyl methacrylate) standards (Agilent EasyVials) were used for calibration between 955,000 and 550 g mol^–1^. Analyte samples were filtered through a nylon membrane with 0.22 μm pore size before injection. The experimental molar mass (*M*_n_, SEC) and dispersity (*D̵*) values of the synthesized polymers were determined by conventional calibration and universal calibration, respectively, using Agilent GPC/SEC software.

### 2.7 Nuclear Magnetic Resonance Spectroscopy

^1^H NMR and ^13^C NMR APT spectra were recorded on Bruker AVANCE 300 and 400 spectrometers (300 MHz and 400 MHz, respectively). Deuterated chloroform, deuterated water, and dimethyl sulfoxide-*d*_6_ were used as solvents for all measurements. Data analysis was performed using MestReNova.

### 2.8 Dynamic Light Scattering Measurements

Copolymers were dissolved in 1 mL deionized water at a concentration of 1024 μg mL^–1^, and the samples filtered with a nylon filter (2 μm). Measurements were performed in polystyrene cuvettes at 37 °C with an Anton-Paar Litesizer 500.

### 2.9 UV–Vis Measurements

Turbidity analyses for the determination of the transition temperature of each sample were performed using an Agilent Technologies Cary 100 UV–Vis spectrophotometer equipped with an Agilent Technologies Cary temperature controller and an Agilent Technologies 6 × 6 multicell block Peltier. The measurements were performed using Suprasil quartz cuvettes (Hellman, 100-QS, light path = 10.00 mm) filled with 2 mg mL^–1^ solution of each polymer in PBS. For each sample, two heating/cooling cycles between 25 and 60 °C were performed with a temperature gradient of 1 °C/min at λ = 633 nm. All data points were recorded using the Cary WinUV software.

### 2.10 Minimum Inhibitory Concentration Assay

Minimum inhibitory concentrations (MICs) were determined according to the standard Clinical Laboratory Standards Institute (CLSI) broth microdilution method (M07-A9-2012).^39^ A single colony of bacteria in agar plates was chosen and dissolved in fresh cationic adjusted Mueller–Hinton broth (caMHB). The concentration of the bacterial cells was adjusted by measuring the optical density at 600 nm (OD_600_) to obtain a 0.5 Mackfarland equivalent, thus reaching a bacterial concentration of approximately 1 × 10^8^ colony forming unit per mL (cfu mL^–1^). The solution was further diluted 100-fold to obtain a concentration of 1 × 10^6^ cfu mL^–1^. Polymers were dissolved in respective media, and 50 μL of each polymer solution was added to micro-wells followed by the addition of the same volume of bacterial suspension, resulting in a final bacterial density of 5 × 10^5^ cfu mL^–1^. The micro-well plates were incubated at 37 °C for 18 h. Then, the growth was evaluated by addition of 10 μL resazurin dye to each well leading to a final concentration of 0.5 mg mL^–1^. The plates were incubated for 30 min at 37 °C, and a noticeable change of color could be observed where bacteria cells grew (pink color) while the suspension remained blue in wells with non-detectable growth. Resazurin was prepared at 0.5 mg mL^–1^ stock in PBS, and the solution was filter sterilized (0.22 μm filter). The solution was stored at 4 °C and covered in foil for a maximum of 2 weeks after preparation. The protocol was followed as described before by Elshikh et al.^40^

### 2.11 Hemolysis Assay

Sheep RBCs were prepared by washing with PBS via centrifugation (4500*g* for 1 min) until the supernatant was clear. Polymers were dissolved in PBS up to 1024 μg mL^–1^ as the highest concentration. A solution of 1% Triton X-100 was used as a positive control, and a solution of PBS was used as a negative control. 100 μL of 6% (v/v) of RBCs in PBS was added to each well of a 96-well plate. Then, 100 μL of each polymer solution was added to make up a total volume of 200 μL and was mixed before being incubated at 37 °C for 2 h. The 96-well plates were centrifuged at 600*g* for 10 min, and 100 μL of the supernatant was transferred to a new plate. The absorbance at 540 nm was measured and normalized with the positive and negative control. Positive control (Triton X-100) was used as 100% cell lysis, and negative control (PBS) as 0%.

### 2.12 Hemagglutination Assay

Sheep RBCs were prepared by washing with PBS via centrifugation until the supernatant was clear. 50 μL of 6% (v/v) of RBCs in PBS was added to each well of a “U” bottom 96-well microplate. Polymers were dissolved in PBS up to 1024 μg mL^–1^ as the highest concentration. Concanavalin A (0.05 mg mL^–1^) solution was used as a positive control, and PBS was used as a negative control. Then, 50 μL of each polymer solution was added to make up a total volume of 100 μL and was mixed before being incubated at 37 °C for 1 h. After the incubation period, the hemagglutination was assessed by visually comparing the treatments wells with the control wells.^41^ For the light microscopy images, the assay was performed in a 24-well plate only at the highest polymer concentration and directly imaged after incubation.

### 2.13 SEM Sample Preparation

A single colony of bacteria in agar plates was chosen and dissolved in 5 mL of fresh caMHB and incubated overnight at 37 °C. The culture suspension was then diluted down to an OD_600_ value of 0.1 and placed back in the incubator for 3 h. 1 mL of this bacterial solution was incubated in the presence of 1 mL of a polymeric treatment dissolved in caMHB (at 0.5 MIC, MIC and 2× MIC) at 37 °C for 1 h. Then the cells were pelleted by centrifugation at 12,000 rpm for 2 min, followed by three washes with sterile PBS. On the final wash, the cells were resuspended in 400 μL of PBS.

In the meantime, 12 mm diameter circular coverslips were incubated with 50 μL of poly-lysine in a 24-well tissue culture plate. After 15 min, the poly-lysine solution was removed, and the coverslips were left to dry. 50 μL of the bacterial cell suspension was added to the coverslips and left to incubate for 30 min at room temperature. Then, the excess volume was removed, and the cells were fixed overnight with a 2.5% glutaraldehyde solution in PBS at 4 °C. After fixation, the 2.5% glutaraldehyde solution was discarded, and the coverslips where rinsed three times with PBS. In the last step, the coverslips were moved to clean wells, and dehydration was performed using an ethanol gradient (20, 50, 70, 90, 100, and 100% again) for 10 min at each concentration. After complete dehydration, the coverslips were moved to clean wells and were incubated with 0.5 mL of HDMS as a drying agent for 30 min; the HDMS solution was then discarded, and the coverslips were moved to clean wells and left to dry in a flow laminar cabinet for 30 min. Next, copper tape was added to scanning electron microscopy (SEM) sample holders, and the coverslips were placed on top. Finally, the samples were sputtered with carbon and immediately analyzed using a Zeiss Gemini Scanning Electron Microscope equipped with an InLens detector, at a voltage of 1 kV.

### 2.14 Biofilm Prevention Assay on Coupon Discs

Briefly, an overnight culture of *P. aeruginosa* ATCC 15442 was prepared by inoculating a single colony from an LB plate into 10 mL of tryptic soy broth (TSB) and grown 24 h at 37 °C with shaking (∼125 rpm). Then, the overnight culture was adjusted to a 0.05 McFarland, equivalent to 1 × 10^7^ cfu mL^–1^. Sterile coupon discs were placed in 12-well plates, and 3 mL of the polymeric solution at 0.5× MIC and MIC in PBS were added. Immediately after, 1 mL of bacterial solution was added, and the plates were incubated at 37 °C for 24 h with shaking (125 rpm). After 24 h, the coupons were rinsed twice with PBS and transferred to falcon tubes with 10 mL Dey Engley neutralizing solution, and they were sonicated for 30 min. Then, each sample was serial diluted in PBS plated in TSA in duplicates (50 μL onto each half of the plate). The plates were incubated at 37 °C and counted. Three coupon discs were used for each condition tested, and three different experiments using three different overnight bacterial cultures were performed.

### 2.15 24 h CDC Biofilm Assay

The Center for Disease Control (CDC) bioreactor was used to establish 24 h biofilms of *P. aeruginosa* ATCC 15442, following an adapted version of ASTM 2871-19. Briefly, an overnight culture of *P. aeruginosa* ATCC 15442 was prepared by inoculating a single colony from an LB plate into 10 mL of TSB and grown 24 h at 37 °C with shaking (∼125 rpm). Then, the overnight culture was adjusted to a 0.5 McFarland, equivalent to 1 × 10^8^ cfu mL^–1^, and 1 mL of this solution was incubated into the CDC bioreactor previously filled with 300 mL of TSB. The bioreactor was incubated in batch phase on a magnetic stir plate (125 rpm) for 24 h at room temperature. After 24 h, the rods were washed twice in PBS and individual coupon discs were placed into 12-well plates. Then, the coupons were exposed to polymeric solution at 4× MIC (512 μg mL^–1^) in PBS, and untreated controls were exposed to PBS. After 24 h treatment, the coupons were transferred to falcon tubes with 10 mL Dey Engley neutralizing solution, and they were sonicated for 30 min. Then, each sample was serial diluted in PBS plated in TSA in duplicates (50 μL onto each half of the plate). The plates were incubated at 37 °C and counted. Three coupon discs were used for each condition tested, and three different experiments using three different overnight bacterial cultures in three different CDC bioreactors were performed.

## 3 Results and Discussion

### 3.1 Star Polymer Design and Synthesis

**Scheme 1:**
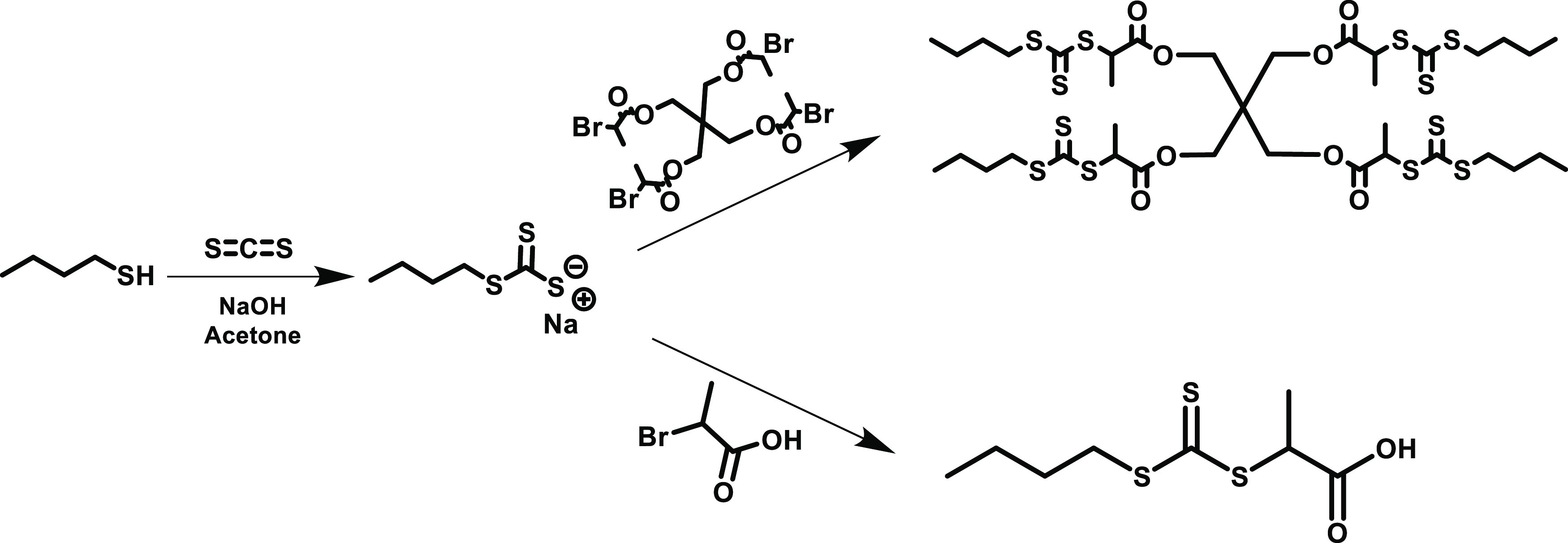
Synthesis of PABTC vs Four-Armed CTA

The star copolymers for this study were synthesized through a core-first approach using reversible addition–fragmentation chain transfer (RAFT) polymerization, in which the arms of the star polymer are grown from a multifunctional RAFT agent. Based on previous publications on this monomer system,^25^ we decided to use 2-((butylthio)-carbonothioyl) thio propanoic acid (PABTC), a trithiocarbonate CTA. In order to synthesize a four-armed star polymer with this CTA, four PABTC units were linked together by ester bonds via a pentaerythritol core (Scheme 1). All linear copolymer controls were also synthesized by RAFT polymerization using PABTC as the CTA.

Acrylamides were chosen as the monomer class for this study as they do not hydrolyze in aqueous solution, and this enables the synthesis of complex and well-defined materials in a straightforward manner by RAFT polymerization.^17^ In particular, their fast rate of propagation enables the use of low radical initiator concentrations during reaction and therefore limits side reactions such as star–star coupling.^42^ Furthermore, the resulting polymer structures have been shown to be resistant to degradation by enzymes^25^ and to be stable under the acidic conditions needed to remove the protecting groups of the side chains. For the cationic unit, aminoethyl acrylamide (AEAm) was used as a lysine-mimic (an amino acid commonly found in AMPs),^13^ which has shown good properties in antimicrobial polymers.^43^ To prevent aminolysis of the trithiocarbonate group of the CTA during polymerization, the primary amine of AEAm was protected with a Boc-group, which was removed post-polymerization to yield the final cationic polymer. The apolar segments were based on NIPAm, a good mimic of the amino acid leucine commonly found in AMPS, and which is known to lead to low hemolytic activity according to previous studies.^23,25,44^ p(NIPAm) is a thermoresponsive polymer with an LCST of approximately 32 °C and can be defined as a hydrophilic polymer as it is water soluble at room temperature.^45^ However, the apolar isopropyl side chain confers an apolar character, and overall, NIPAm can be defined as an amphiphilic monomer with apolar and polar groups.^46^ A hydrophobic monomer such as butyl or hexyl acrylate can lead to copolymers with a higher antimicrobial activity and a better ability to disrupt and penetrate a bacterial membrane.^47^ However, this can also lead to an increased hemolytic activity,^44^ self-assembly of the polymers into micelles which can reduce the interaction of the hydrophobic units with the lipid bilayer,^26^ and reduced solubility in aqueous media.

Consequently, NIPAm copolymerized with a cationic co-monomer such as amino ethyl acrylamide leads to a final compound with characteristics of an amphiphilic copolymer, which has been shown to result in promising antimicrobial activity and low toxicity.^23−25^ Furthermore, the LCST of p(NIPAm) can be increased through copolymerization with hydrophilic or cationic monomers, leading to water-soluble polymers at physiological temperatures of 37 °C.^48^

**Scheme 2:**
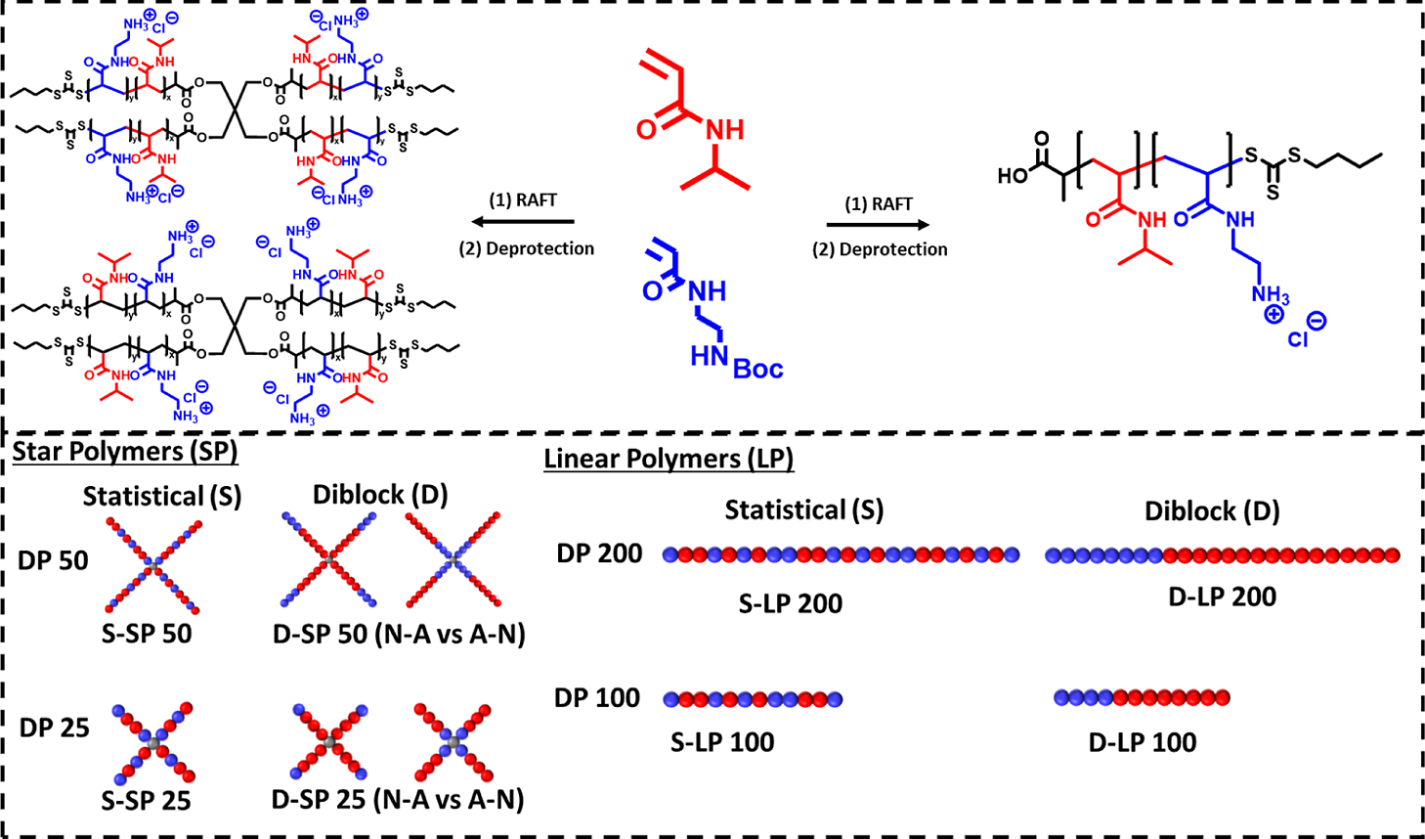
Structure and Overview of the Six Star and Four Linear Copolymers S and D describe a star and a diblock copolymer, respectively; SP and LP describe a star copolymer and a linear copolymer, respectively, followed by the target DP for each polymer (25/50 for stars, 100/200 for linear). For diblock stars, the order of the polymer blocks is abbreviated by N-A vs A-N (pNIPAm-*b*-pAEAm vs pAEAm-*b*-pNIPAm). As an example, the diblock star copolymer with a DP 25 and pNIPAm core is abbreviated as D-SP25 (N-A).

Monomer distribution and segmentation have been shown to influence the structure–activity relationship of linear SNAPs.^25^ We synthesized s-SNAPs with statistical arms and diblock copolymer arms, the latter with different sequences in apolar and polar segments (Scheme 2). Indeed, chain extension of the pNIPAm chains with AEAm led to a pNIPAm core and pAEAm outer shell (N-A), and chain extension of pAEAm with NIPAm led to a pAEAm core and pNIPAm outer shell (A-N).

The length of the copolymer arms was also varied, with a targeted degree of polymerization (DP) of 25 and 50. Equivalent linear copolymers were, therefore, synthesized with a DP of 100 and 200, resulting in a total of six star polymers and four linear polymers (Scheme 2). The ratio of cationic units was kept consistent at 30%, independent of the molecular weight, based on a previous study on these monomers that has shown that this ratio leads to optimal antimicrobial activity and hemocompatibility.^25^

**Figure 1:**
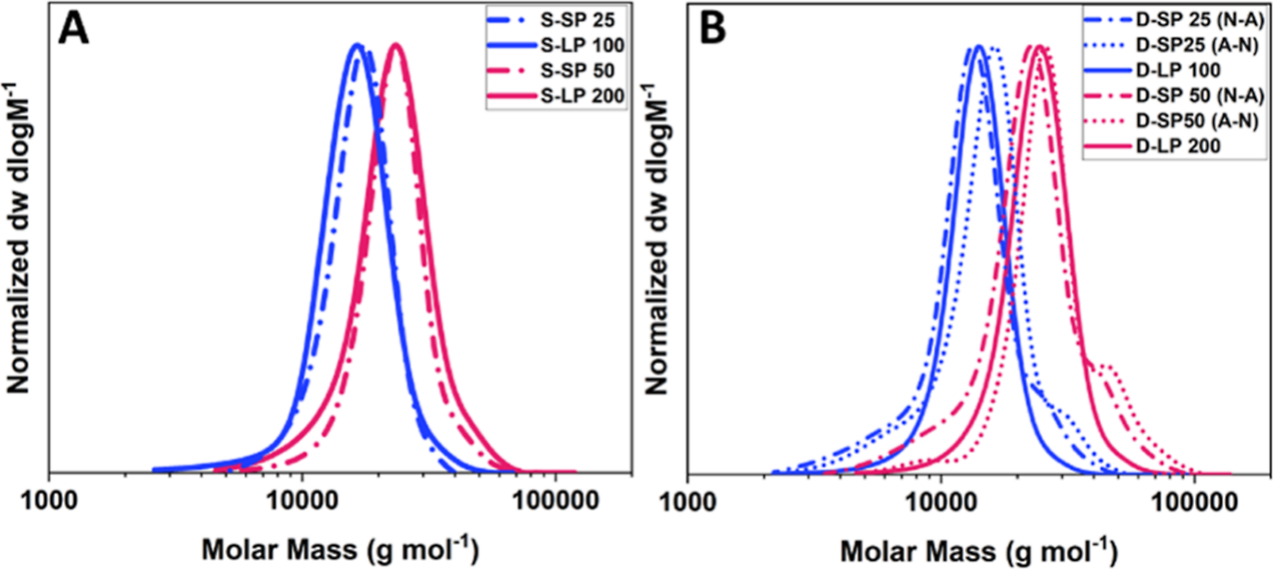
SEC traces of four statistical copolymers (A) and diblock copolymers (B) (DMF GPC, PMMA standard).

The linear polymers were successfully synthesized, with dispersities below 1.3 at full conversion (Table 1). The SEC traces show a similar hydrodynamic volume for linear polymers compared to the equivalent star polymers. We observed a small high-molecular-weight shoulder for the diblock star copolymers, which suggests star–star coupling (Figure 1). This was observed both after chain-extension of the NIPAM block with the BocAEAm monomer (Figure S21) and after chain extension of the BocAEAm block with NIPAm. Star–star coupling arises from termination reactions by combination and irreversible chain transfer, and it is expected to be more preponderant at full conversion. However, since the dispersity is kept below 1.3 for all materials and to keep consistent with a realistic synthetic process that does not require monomer removal after polymerization, the materials were used as obtained.

**Table 1:** SEC (DMF GPC, PMMA Standard) and NMR (400 MHz CDCl_3_) Results for Four Linear and Six Star Copolymers

polymer	conversion (%)	DP_NMR_	*M*_nNMR_ (g mol^–^^1^)	*M*_nSEC_^a^ (g mol^–^^1^)	*M*_nMALS_^b^ (g mol^–^^1^)	*Đ*
S-SP 25	88	22	17,000	15,400	16,200	1.11
S-SP 50	90	45	29,700	21,600	31,000	1.13
S-LP 100	99	100	14,600	14,800	12,500	1.14
S-LP 200	99	200	28,900	20,700	25,000	1.16
D-SP 25 (N-A)	99	25	15,400	11,600	13,000	1.23
D-SP 50 (N-A)	99	50	29,700	19,600	27,100	1.25
D-SP 25 (A-N)	99	25	15,400	14,500	16,900	1.22
D-SP 50 (A-N)	99	50	29,700	26,400	29,500	1.18
D-LP 100	99	100	14,600	13,000	12,700	1.10
D-LP 200	99	200	28,900	22,400	25,600	1.11

a Obtained through single detection GPC.

b Obtained through triple detection GPC with universal calibration.

After synthesis, the Boc-protecting group was removed by treatment with TFA, and the materials were dialyzed to change the TFA counterion to chloride.

For all SNAPs, dynamic light scattering (DLS) measurements were conducted at a concentration of 1.024 mg mL^–1^ at 37 °C and showed that at this concentration, no aggregation or self-assembly occurs. The measured count rates were too low; consequently, no meaningful reading for their size could be obtained. This is in agreement with previous studies on p(NIPAm-*co*-AEAm), which showed no self-assembly as the cationic primary amine group appears to prevent self-assembly even for block copolymers.^24,25^

As pNIPAm is known to have an LCST of around 32 °C, UV–vis measurements were conducted at 2 mg mL^–1^ for all star copolymers (Figure S19). Previously, Garcia Maset et al. have shown that similar linear diblock copolymers p(NIPAm-*co*-AEAm) are not thermoresponsive in PBS at 37 °C.^23^ The star copolymers showed the same effect as their linear counterparts, as no cloud points were observed within the tested temperature range of 25–60 °C.

### 3.2 Structure–Activity Relationship

#### 3.2.1 s-SNAPs Versus SNAPs: Antimicrobial Activity

To determine if the star architecture affects the antimicrobial activity of the copolymers, the MICs of each compound (using the cell viability dye resazurin) were determined (Figure S22). Two bacterial species were selected as representative models (Table 2): the Gram-negative *P. aeruginosa* PA14, a highly virulent laboratory strain,^49^ and the Gram-positive *S. aureus* USA300, a methicillin-resistant strain and strong biofilm former.^50^

**Table 2:**
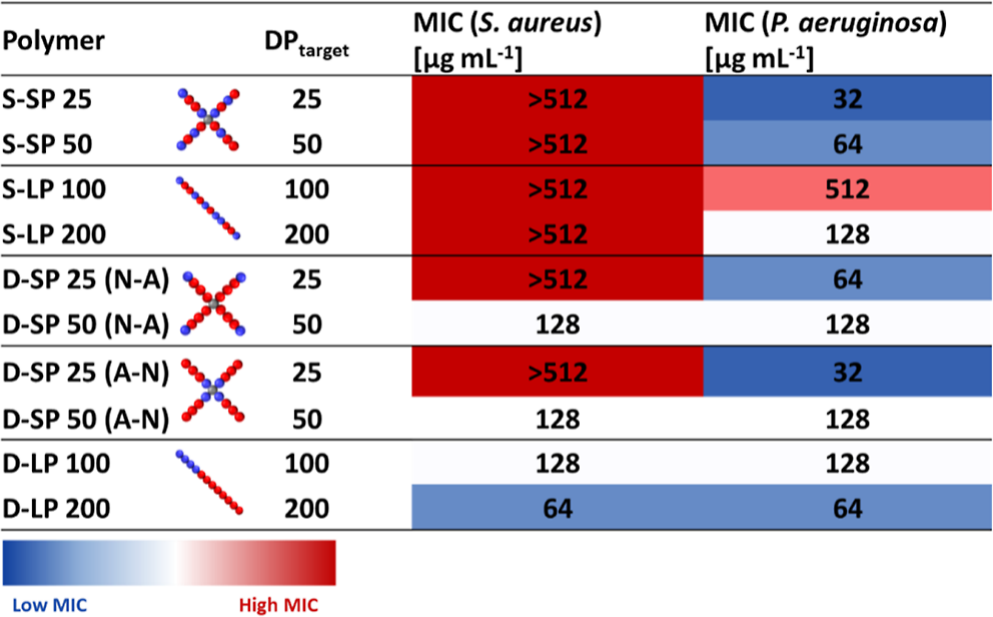
Antimicrobial Activity of the Copolymers^a^

a MICs values expressed in μg mL^–1^ of the copolymers tested in caMHB against *S. aureus* USA300 and *P. aeruginosa* PA14. The heatmap shows low MIC (high activity) in blue and high MIC (low activity) in red. Three independent biological experiments were performed on different days, and the highest MIC value was reported.

To discuss the influence of the star architecture on the antimicrobial activity, a direct comparison can be made with the linear copolymer equivalents (Table 2). The molecular weight of linear and star copolymers can be comparable, although the presence of the pentaerythritol core (Scheme 1) in the four-armed CTA results in a slightly increased molecular weight for the stars.

Only four copolymers, D-LP100, D-LP200, D-SP50 (N-A), and D-SP50 (A-N), showed antimicrobial activity against *S. aureus* USA300. Both linear diblock copolymers (D-LP100 and D-LP200) have equal MIC values against both strains, while a statistical monomer distribution in linear and star copolymers led to inactivity against *S. aureus* USA300. D-SP50 (N-A) and D-SP50 (A-N) are the only star copolymers with antimicrobial activity toward the Gram-negative and Gram-positive bacteria selected in this study; however, they are less active compared to the linear copolymer equivalent D-LP200. Changing the architecture from linear to a star copolymer does not appear to have a positive influence on the antimicrobial activity for *S. aureus* USA300. It might be possible that the larger star architecture has a decreased ability to penetrate the peptidoglycan cell wall of *S. aureus*, thus explaining its lower activity.

Interestingly, a selectivity toward *P. aeruginosa* PA14 can be observed, as all star copolymers except for D-SP50 (N-A) and D-SP50 (A-N) show no activity toward *S. aureus* USA300. This could point to an overall selectivity toward Gram-negative strains for these materials; however, this would have to be confirmed through testing against a variety of Gram-negative vs Gram-positive species.

All 10 copolymers were active toward PA14 *P. aeruginosa*. As observed by comparing the MIC values of the star copolymers to their linear equivalent, the most striking difference occurred in the statistical copolymer library for the S-SP25 copolymer with a fourfold reduction compared to the linear copolymer S-LP100 (Table 2). The influence of the architecture on antimicrobial activity decreased with a higher molecular weight with, for example, only a onefold difference in MIC for S-SP50 vs S-LP200.

Overall, molecular weight affected the antimicrobial activity of the statistical linear copolymers, but it had a minimal influence on the antimicrobial performance of the diblock linear and the star copolymers against *P. aeruginosa* PA14. A previous study on how molecular weight affects antimicrobial activity in star copolymers has shown that there is a threshold, above which an increase in the length of the arms no longer improved the antimicrobial activity.^38^ Furthermore, for D-SP50 (N-A) and D-SP50 (A-N), changing the positioning of the cationic block within the structure did not have any effect on the antimicrobial activity. This suggests that above a certain molecular weight, the changes in monomer positioning within these polymers do not affect the structure–activity relationship. Therefore, for our star copolymers, arms with a DP 25 might be the threshold above which activity cannot be further enhanced.

The smaller star copolymers S-SP25 and D-SP25 showed the lowest overall MIC values against *P. aeruginosa* PA14 and therefore showed the most potent antimicrobial activity. Further improvement for the D-SP25 stars was observed when the cationic block was located on the core of the star D-SP25 (A-N). This suggested that the apolar block on the outside of the star structure slightly increased the antimicrobial activity for the smaller star copolymers. We theorize that the apolar arms could benefit the disruption of the bacterial membrane. Additionally, the cationic charges in the center of the star structure could be more protected by the apolar blocks to avoid interaction with proteins or cations/anions present under biological conditions that have been shown to have a negative effect on the antimicrobial activity of AMPs.^51^

#### 3.2.2 Hemocompatibility of Star Versus Linear Copolymers

SNAPs and AMPs have been shown to exhibit toxicity against mammalian cells, which has reduced their biological applications.^52^ We investigated the hemocompatibility of the copolymers by determining the hemolytic activity (**Hc**_**10**_) and hemagglutination (**C**_**H**_) (Table 3) against sheep RBCs (Figures S23 and S24).

**Table 3:**
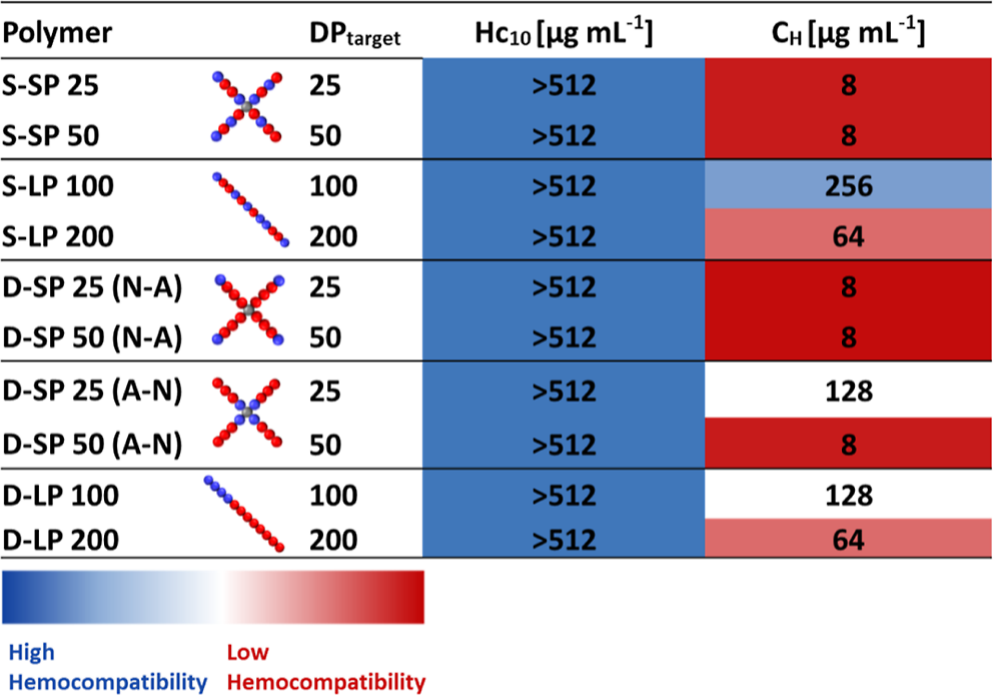
Hemolytic Activity (**Hc**_**10**_—Minimum Concentration Which Induces Lysis of 10% of RBC), Hemagglutination (**C**_**H**_—Minimum Concentration of the Antimicrobial Agent or Treatment at Which 10% of RBCs Aggregated)^a^

a Three independent biological experiments were performed on different days, and the highest toxicity value was reported.

All copolymers were not found to be hemolytic within the tested concentration range (8–1024 μg mL^–1^). Five out of the six star copolymers induced aggregation at 8 μg mL^–1^, regardless of their monomer composition or length of arms. The only exception was observed for the D-SP25 (A-N) compound, which induced aggregation at a 16-fold increased concentration (128 μg mL^–1^) in comparison with the rest of the s-SNAPs.

For the larger star copolymers D-SP50 (N-A) vs D-SP50 (A-N), the positioning of the cationic block did not appear to have an effect on hemagglutination. We theorize that the increased molecular weight of these stars “overshadows” the effects of changing the positioning of the cationic units. Molecular weight appears to increase aggregation overall within this polymer library, as the linear polymers S-LP200 and D-LP200 aggregate at a lower concentration compared to the LP100 compounds, regardless of their monomer distribution. Polymers with a higher molecular weight have an overall higher number of cationic units within the structure. It has been shown in previous publications that increasing the number of cationic units within a polymer can increase hemagglutination.^53^

We, therefore, conclude that the positioning of the cationic block in D-SP25 (N-A) vs D-SP25 (A-N) structures greatly influenced the aggregation of RBC. Increasing the molecular weight of the stars reduced the influence of changing the cationic block position, as with longer arms, the cationic units are possibly more exposed to cells.

Overall, star architectures with a higher molecular weight and with cationic units statistically distributed or on the outside of the structures were most effective at causing aggregation of RBCs. This indicates that cationic units, in addition to the change in architecture, play a role in inducing aggregation for these compounds. In addition, to visibly observe the hemagglutination caused by the star copolymer, samples were taken directly from the wells after the hemagglutination assay and imaged with light microscopy (Figure S25).

### 3.3 Selectivity of Star Versus Linear Copolymers

To highlight the importance of determining both hemolytic activity and hemagglutination values, the selectivity index was calculated for both **C**_**H**_ and **H**_**C10**_ values. The ratio between antimicrobial activity and cytotoxicity was calculated, as can be seen in Table 4. As no polymers induced hemolysis within the measured concentration range (8–1024 μg mL^–1^), the resulting selectivity index was much higher compared to the index calculated with hemagglutination values (Table 4). For the copolymers that did not show antimicrobial activity within the measured concentration range, no MIC could be determined; therefore, no selectivity value was calculated.

**Table 4:**
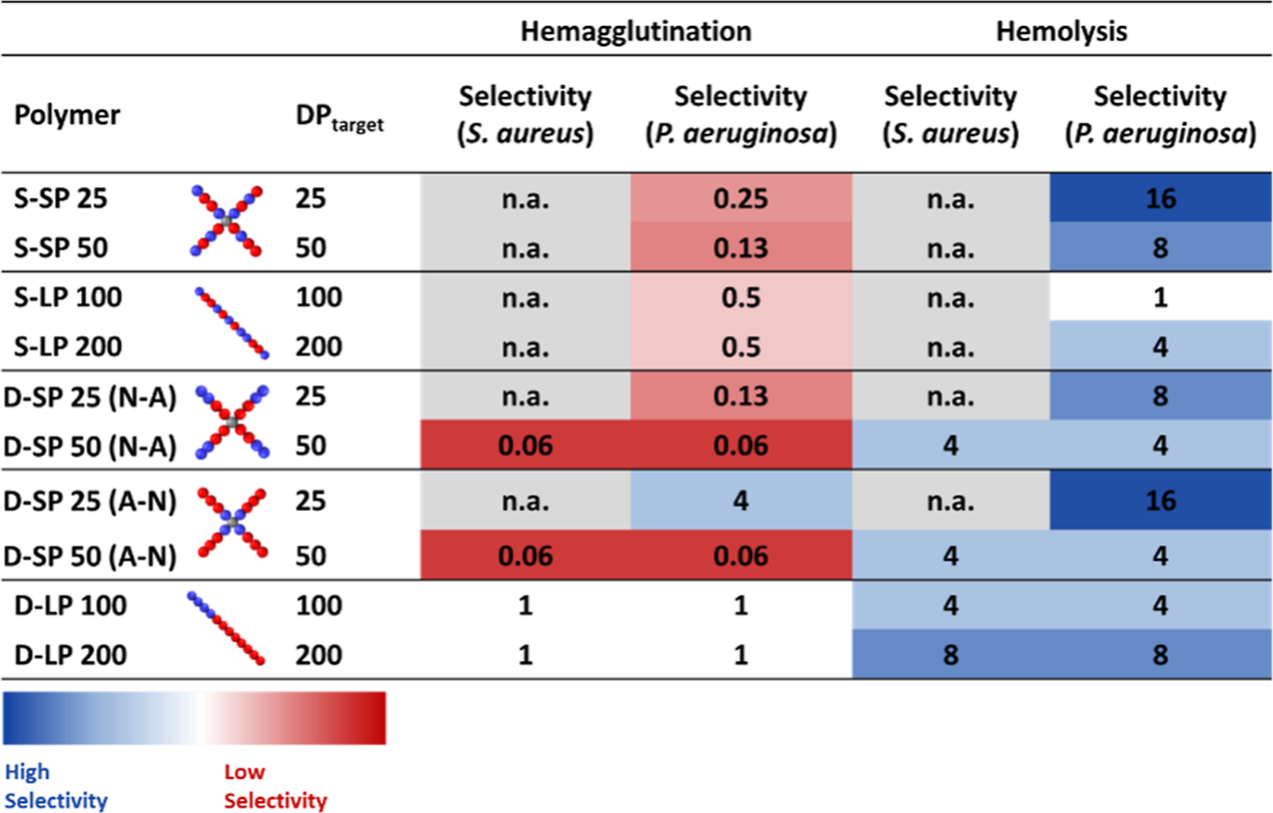
Selectivity [Hemocompatibility Value (**C**_**H**_ or **H**_**C10**_ Divided by MIC)] Values for *S. aureus* USA300 and *P. aeruginosa* PA14^a^

a Selectivity for both **C**_**H**_ and **H**_**C10**_ were calculated to underline the importance of determining both hemocompatibility values.

A selectivity value ≤1 indicates that the cytotoxicity is greater or identical to the MIC, resulting in a polymer that is causing lysis or aggregation of RBCs within the antimicrobial active concentration range. A selectivity value >1 is a good indication of an efficient antimicrobial polymer that will inhibit bacterial growth without causing RBC lysis or aggregation at the MIC.

Five star polymers have a low selectivity for hemagglutination due to their low **C**_**H**_ values of 8 μg mL^–1^, and the linear copolymers achieve a maximum index of 1. The only polymer with a higher selectivity value of 4 with regard to the hemagglutination was the D-SP25 (A-N) compound. In contrast, taking into account the hemolysis values and the MICs against *P. aeruginosa* PA14, the star polymer architecture improved the selectivity indexes. For example, both S-SP25 and D-SP25 (A-N) showed a selectivity index of 16 in comparison with their linear equivalents S-LP100 and D-LP100 with an index of 1 and 4.

The most promising compound of this study is D-SP25 (A-N), as it is the only copolymer that showed selectivity toward *P. aeruginosa* PA14 over RBCs for both hemolysis and hemagglutination.

### 3.4 Aggregation Effect of Star Architecture Copolymers against *P. aeruginosa* PA14

Overall, the star polymer architecture induced hemagglutination at very low concentrations, except for the star copolymer with the cationic block in the core [D-SP25 (A-N)]. We, therefore, hypothesized that the cationic units introduced with the AEAm monomer are a driving factor for the aggregation of RBCs, and when located at the core of the smaller star copolymer, this effect is suppressed.

**Figure 2:**
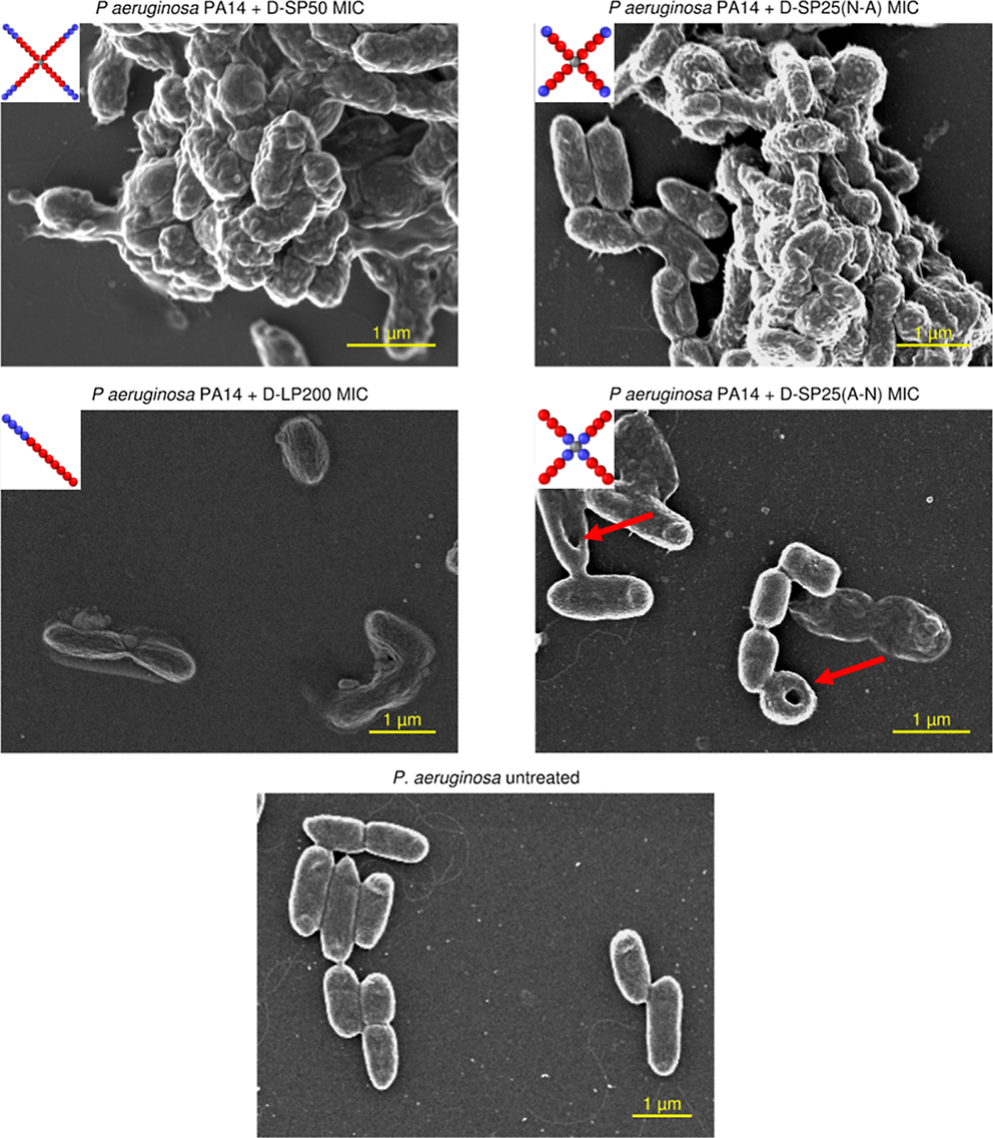
Scanning electron micrographs of *P. aeruginosa* PA14 exposed to caMHB (untreated control), D-SP50 (N-A), D-LP200, D-S25 (A-N), and D-SP25 (N-A) for 1 h at MICs. The red arrows denote the “pore formation” in*P. aeruginosa* PA14 cells after the polymeric treatment of D-S25 (A-N).

We investigated whether the stars could induce aggregation of bacterial cells and whether the position of the cationic block also influenced this aggregation. We selected four polymer candidates in total: the star copolymer D-SP50 (N-A) and its linear counterpart D-LP200 to study the influence of linear versus star architecture, and the star copolymers D-SP25 (A-N) and D-SP25 (N-A) to study the effect of the position of the pAEAM block. *P. aeruginosa* PA14 was incubated with the polymeric treatments at 0.5× MIC, MIC, and 2× MIC for 1 h, and the effect on the cell morphology and aggregation was investigated using SEM (Figure 2). *P. aeruginosa* PA14 grown in caMHB without treatment was used as a control.

Both the linear and star copolymers appear to affect the bacterial cell morphology through disrupted membranes at all three measured concentrations. However, there were clear differences observed for star versus linear copolymer, as after incubation with D-SP50 (N-A), the bacterial cells formed aggregates for all three concentrations, while the linear counterpart D-LP200 did not have this effect on the bacteria. This different phenotype effect observed for the star versus the linear copolymer counterparts in the SEM images indicates a potential difference in the mechanism of action between the star and linear copolymer architecture. The same aggregation effect toward *P. aeruginosa* PA14 cells was observed for D-SP25 (N-A) and S-SP50 (Figures S26 and S27), demonstrating that a change in molecular weight and monomer distribution does not seem to affect the bacterial aggregation effect of the star architecture.

However, D-SP25 (A-N) did not cause aggregation at any of the three concentrations tested (Figure S26). Damaged cells were observed, and potential pore formation was observed for some of the bacterial cells (red arrows in Figure 2), which was not found for any of the other polymer samples investigated in this study.^54^ D-SP25 (A-N) is the only polymer with a selectivity index above 1 for both hemolysis (selectivity index 4) and hemagglutination (selectivity index 16) toward *P. aeruginosa* PA14. This points to the hypothesis that by switching from a linear to a star polymer architecture with the hydrophobic pNIPAm block on the outside, the aggregation effect could be reduced both for RBCs and bacterial cells while maintaining high activity and membrane disruption toward bacterial cells.

The aggregation observed in *P. aeruginosa* resembles a biofilm structure. Biofilms can be defined as 3D-structured bacterial communities composed of bacterial cells and a protective scaffold of an extracellular polymeric matrix. The EPS matrix, also termed matrixome, is composed of exopolysaccharides, proteins, nucleic acids, and lipids, acting as a protective scaffold.^55^ We hypothesized that the copolymers caused a direct effect on *P. aeruginosa* cells, inducing aggregation. However, the possibility that the copolymers induce biofilm formation, especially at concentrations below the MIC, needed to be investigated.

We hypothesized that if bacterial aggregation occurs, even in dead cells after polymeric treatment, the process is most likely to be induced by a “crosslinking” effect through the star polymeric structure. On the contrary, if the aggregation effect was lost after the polymeric treatment of dead bacterial cells, biofilm formation might more likely be the process behind the aggregation observed. Therefore, *P. aeruginosa* PA14 was heat-inactivated and incubated with the star copolymer D-SP50 (N-A) and its linear equivalent followed by SEM imaging (Figure S30).

Our results showed that the dead bacterial cells in the presence of D-SP50 (N-A) form aggregates, while the linear polymer treatment D-LP200 did not show any aggregation (Figure S30). This observation proves that the aggregation effect is solely related to the architecture of the star copolymers, and the aggregation effect is directly triggered by the star polymeric architecture instead of biofilm formation.

In order to confirm that the aggregation effect was not dependent on the activity of the s-SNAP toward the Gram-negative strain *P. aeruginosa* PA14 used in this study, we investigated the possible bacterial aggregation effect against *S. aureus* USA300. We selected the copolymers D-SP25 (A-N), D-SP25 (N-A), and D-LP100 to determine whether positioning of cationic units in the smaller star copolymer library has the same effect against both strains.

Therefore, *S. aureus* USA300 was exposed to them for 1 h at their respective MIC prior to SEM imaging (Figure S28). The two D-SP25 compounds did not exhibit an MIC value; therefore, the highest concentration tested (512 μg mL^–1^) was used. We observed an aggregation pattern effect similar to that observed in *P. aeruginosa* PA14. Interestingly, even though D-SP25 (N-A) was not active against *S. aureus* USA300, aggregation was observed. Furthermore, the effect of the cationic block position on aggregation is shown to be consistent for D-SP25 (N-A) vs D-SP25 (A-N) against both strains, as no aggregation was observed for D-SP25 (A-N) or the linear copolymer D-LP100.

### 3.5 Antibiofilm Properties of D-SP25 (A-N) Against *P. aeruginosa* In Vitro Biofilms

The ability of biofilms to resist antibiotic treatments has become a serious concern, and their ability to grow in many-body systems and foreign body surfaces such as implants and catheters makes them a severe issue in medicine.^56^ D-SP25 (A-N) was selected as our lead candidate to investigate its antibiofilm properties, due to its potent antimicrobial activity and good selectivity as well as its membrane-disrupting properties (possible pore formation) as observed in SEM analysis.

We investigated the activity of D-SP25 (A-N) against *P. aeruginosa* ATTC 15442, an environmental strain known to form biofilms, which was first isolated from a water bottle and is routinely used to test disinfectants.^57^ In order to perform this assay, first, a MIC value was determined for this strain, and it was found to be slightly higher (128 μg mL^–1^) compared to that of *P. aeruginosa* PA14 (32 μg mL^–1^).

**Figure 3:**
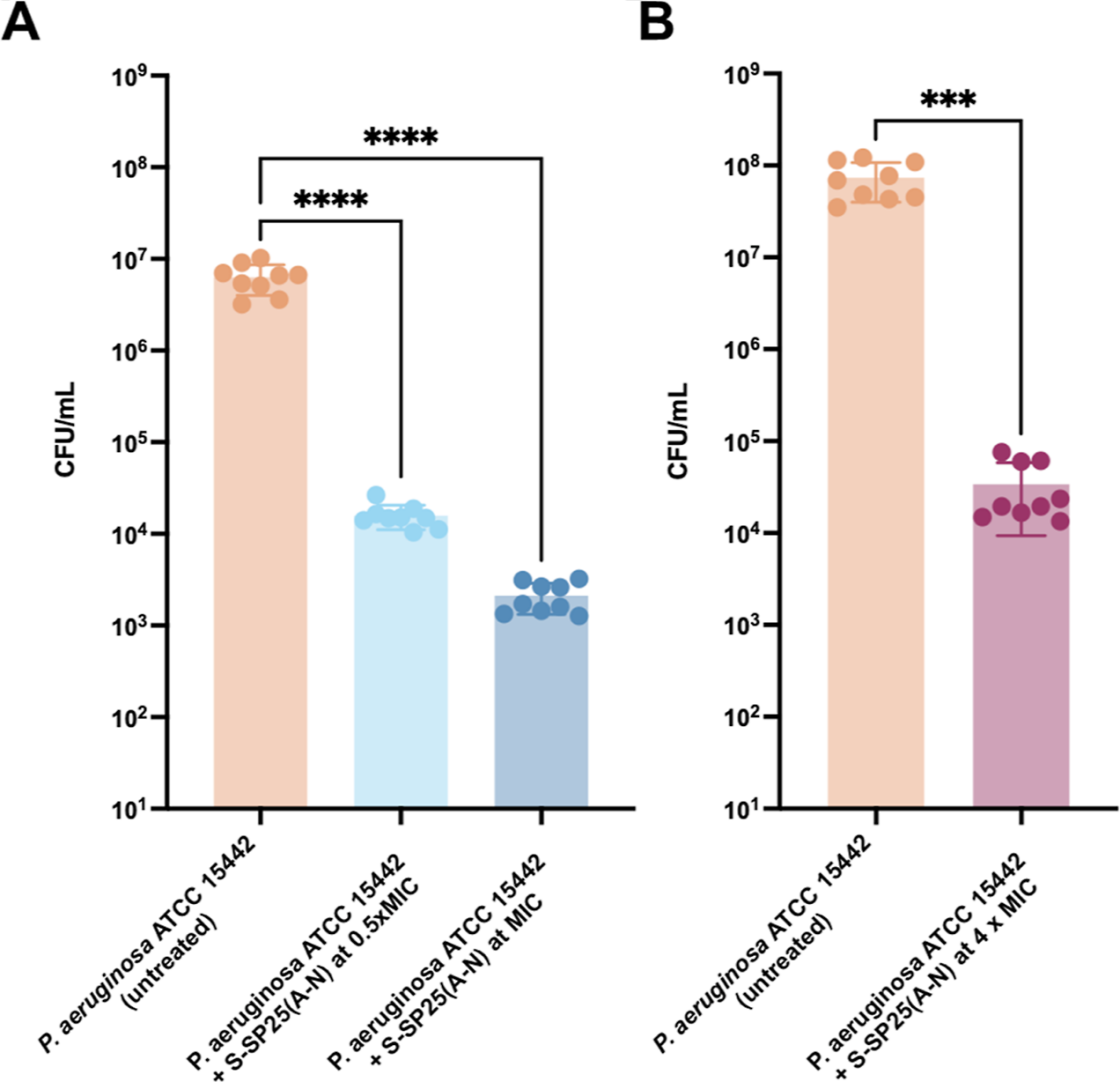
(A) Biofilm prevention assay using rod coupons against *P. aeruginosa* ATCC 15442. An ANOVA and a Dunnett’s test were performed to compare the untreated control with the treatments. (B) Biofilm disruption assay against 24 h biofilms of *P. aeruginosa* ATCC 1554 in the CDC bioreactor. A *t*-test was performed to compare the cfu obtained after the treatment of S-SP25 (A-N) at 4× MIC with the untreated control (PBS). The data were collected from three independent experiments (conducted from different bacterial overnights or days).

Then, we investigated the biofilm prevention properties of the compound against *P. aeruginosa* ATTC 15442 in an *in vitro* CDC biofilm model. The rods were exposed to polymer solution at 0.5× MIC, MIC in PBS, or just PBS (untreated control) and planktonic *P. aeruginosa* ATCC 15442 (0D_600_ = 0.1) in TSB for 24 h. As can be observed in Figure 3A, D-SP25 (A-N) caused a 3-log reduction in the cfu counts at 0.5 MIC and a 4-log reduction in the cfu counts in comparison with the untreated control, evidencing the ability of the compounds to statically reduce the ability of *P. aeruginosa* ATTC 15442 to form biofilms in vitro. The ANOVA test showed significant differences between the untreated controls and the treatments (*F*_2,24_ = 66.84, *p* < 0.001***). Then, a Dunnett’s test was performed to compare the cfu obtained after the treatment with S-SP25 (A-N) at 0.5× MIC and MIC with the untreated control (PBS). Both polymer concentrations showed a significant reduction in cfu in comparison with the untreated control (*p* < 0.0001****).

Similarly, we investigated the biofilm disruption properties of D-SP25 (A-N) against *P. aeruginosa* ATTC 15442 in an *in vitro* CDC biofilm model (24 h biofilm). After 24 h biofilm formation in the CDC bioreactor, the rods were transferred to 12-well plates and exposed to polymer treatment (4× MIC in PBS) and PBS for the untreated controls. D-SP25 (A-N) caused a 3 log-drop reduction in cfu compared to the untreated control, indicating the antibiofilm disruption properties of D-SP25 (Figure 3B). The polymeric treatment showed a significant reduction in the cfu counts in comparison with the untreated control (*t*_8_ = 6.518) (*p* < 0.001***). D-SP25 (A-N) reduced biofilm formation below the MIC and was able to disrupt 24 h biofilms. Therefore, it could be a promising antimicrobial agent to treat biofilm infections, especially for topical applications in wound infections or as surfaces disinfectants for medical devices.

## 4 Conclusions

To conclude, we synthetized multivalent s-SNAPs and their linear equivalents with comparable molecular weight. We investigated the influence of the star architecture on the antimicrobial activity and on the hemocompatibility in comparison with that of their linear copolymer counterparts. Therefore, the impact of the different architectures was assessed independently of size, which is crucial to understand the structure–activity properties of higher-order architectures. We demonstrated that the star architecture has a significant influence on the antimicrobial activity, with an eightfold increase in antimicrobial activity observed for the small statistical star copolymer against *P. aeruginosa* PA14 compared to its linear counterpart. Furthermore, we showed that the star copolymer architecture caused aggregation in RBCs and bacterial cells, while linear copolymers only induce aggregation in RBCs pointing to a difference in the mechanism of action. The aggregation effect in RBCs caused by the star copolymers underlines the importance of performing both hemolysis and hemagglutination assays. Finally, we altered the block position of the cationic block in the star copolymer D-SP25 (A-N), reducing the cell-aggregation effect while showing potent antimicrobial activity. For the D-SP50 (A-N) polymer, this effect was not observed, as the increased molecular weight caused aggregation regardless of block positioning. The electron microscopy analysis revealed a possible pore formation mechanism for D-SP25 (A-N) on *P. aeruginosa* membranes. Varying the block position in the star architecture is a key parameter to tune cell aggregation and antimicrobial activity. Finally, D-SP25 (A-N) showed promising antibiofilm properties against *P. aeruginosa* in a robust *in vitro* biofilm model.

## Supporting Information Available

^1^H NMR spectra of all compounds synthesized in this work, GPC traces of kinetic reaction and chain extensions, images of 96-well plates of MIC and hemagglutination assays, raw data for hemolysis assay, turbidity measurements (UV–vis) of star copolymers, and additional SEM images (PDF)
