# Supplementary material for: Synthetic Star Nanoengineered Antimicrobial Polymers as Antibiofilm Agents: Bacterial Membrane Disruption and Cell Aggregation
Source: Biomacromolecules. 2023 Jun 10;24(7):3073–85. doi: 10.1021/acs.biomac.3c00150 (PMC10336841; doi:10.1021/acs.biomac.3c00150)
Supplement: Supplementary file 1 — bm3c00150_si_001.pdf [file bm3c00150_si_001.pdf]

Supplementary Information

Star Synthetic Nanoengineered Antimicrobial Polymers (s-SNAPs) as antibiofilm agents: bacterial membrane disruption and cells aggregation

Sophie Laroque, †[a] Ramon Garcia Maset,†[a,b] Alexia Hapeshi,[a] Katherine Locock,[c] Sébastien Perrier [a,b,d,] *

[a] *Department of Chemistry, University of Warwick, Gibbet Hill Road, Coventry CV4 7AL, UK.*

[b] *Warwick Medical School, University of Warwick, Coventry, CV4 7AL, UK.*

[c] *CSIRO Manufacturing, Clayton, Victoria 3168, Australia.*

[d] *Faculty of Pharmacy and Pharmaceutical Sciences, Monash University, 381 Royal Parade, Parkville, VIC 3052, Australia.*

Reaction Conditions for Synthesis of Copolymers

Table S1. Reactions conditions for the RAFT polymerization of the statistical linear and star copolymers.

**Polymer**	**S-LP100**	**S-LP200**	**S-SP25**	**S-SP50**
**Monomer 1**	NIPAm	NIPAm	NIPAm	NIPAm
**Monomer 2**	BocAEAm	BocAEAm	BocAEAm	BocAEAm
$\mathbf{DP_{target\ total}}$	100	200	25	50
$\mathbf{DP_{target\ Monomer\ 1}}$	70	140	17.5	35
$\mathbf{DP_{target\ Monomer\ 2}}$	30	60	7.5	15
$\mathbf{m_{monomer1,added}}$ **(mg)**	554	554	277	277
$\mathbf{m_{monomer2,added}}$ **(mg)**	450	450	225	225
$\mathbf{m_{CTA,added}}$ **(mg)**	16.7	8.3	71.2	35.6
$\mathbf{m_{Initiator,added}}$ **(mg)**	0.9	0.5	4.5	2.3
$\mathbf{V_{dioxane,added}}$ **(µL)**	2479	2488	2927	2962
$\mathbf{V_{water,added}}$ **(µL)**	-	-	700	700
$\mathbf{V_{total}}$ **(µL)**	3500	3500	3500	3500
$\mathbf{[CTA]_0/[I]_0}$	20	20	20	20

Table S2. Reactions conditions for the RAFT polymerization of the diblock linear and star copolymers.

Polymer	D-LP100	D-LP200	D-SP25 (N-A)	D-SP50 (N-A)	D-SP25 (A-N)	D-SP50 (A-N)
**Monomer 1**	NIPAm	NIPAm	NIPAm	NIPAm	BocAEAm	BocAEAm
**Monomer 2**	BocAEAm	BocAEAm	BocAEAm	BocAEAm	NIPAm	NIPAm
$\mathbf{DP_{target\ total}}$	100	200	25	50	25	50
$\mathbf{DP_{target\ Monomer\ 1}}$	70	140	17.5	35	7.5	15
$\mathbf{DP_{target\ Monomer\ 2}}$	30	60	7.5	15	17.5	35
$\mathbf{m_{monomer1,added}}$ **(mg)**	679	679	792	792	220	220
$\mathbf{m_{monomer2,added}}$ **(mg)**	375	375	220	220	792	792
$\mathbf{m_{CTA,added}}$ **(mg)**	21	10	102	51	102	51
$\mathbf{m_{Initiator,added}}$ **(mg)**	1.2	0.6	5.6	2.8	5.6	2.8
$\mathbf{V_{dioxane,added}}$ **(µL)**	2423	2435	2386	2437	2386	2437
$\mathbf{V_{total}}$ **(µL)**	3498	3499	3500	3500	3500	3500
$\mathbf{[CTA]_0/[I]_0}$	15	15	15	15	15	15

NMR and IR Spectra

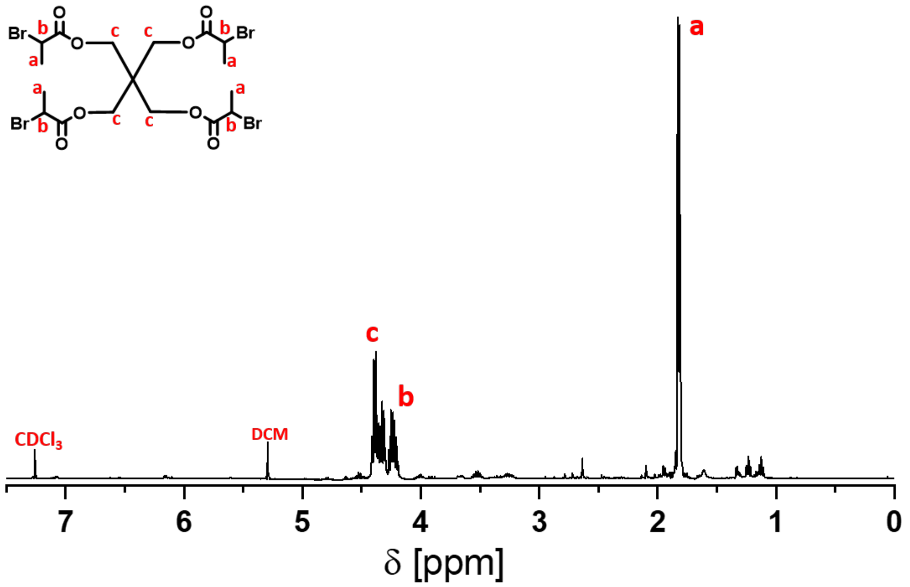

Figure S1: ^{1}H-NMR Spectra of 4-arm CTA precursor (Solvent $CDCl_3$, 300 MHz).

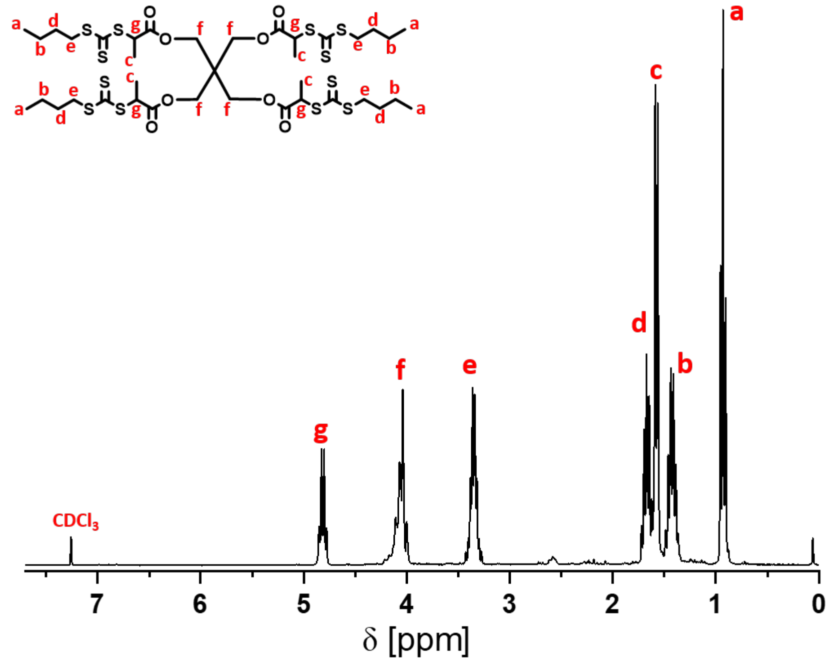

Figure S2: ^{1}H-NMR Spectra of 4-arm CTA (Solvent $CDCl_3$, 300 MHz).

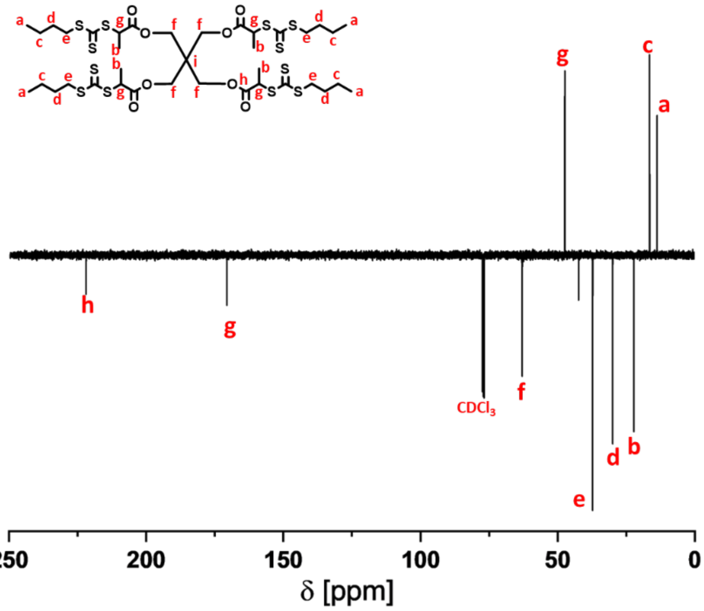

Figure S3: APT ^{13}C-NMR of 4-arm CTA (Solvent $CDCl_3$, 400 MHz).

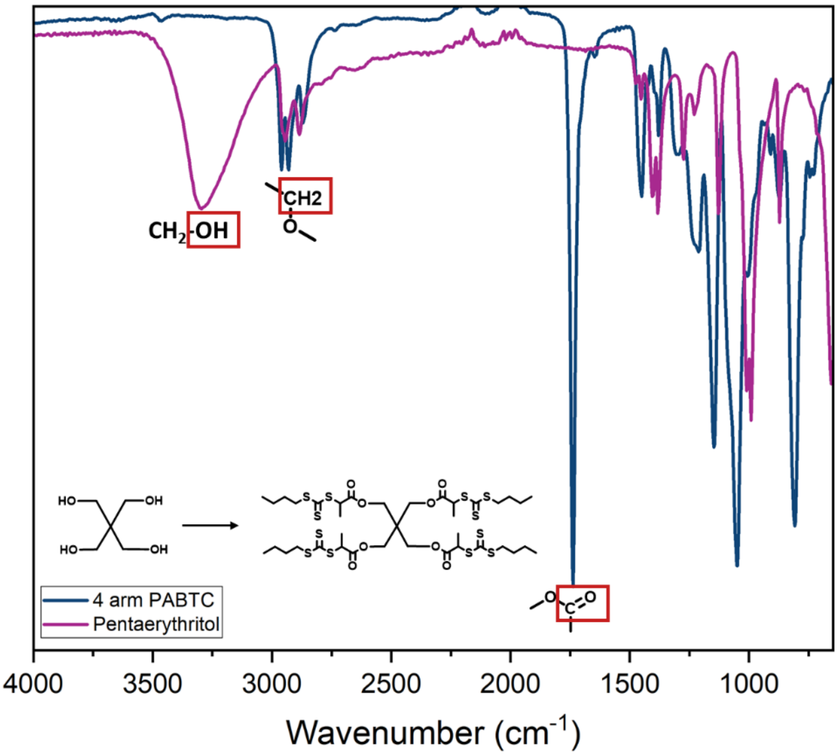

Figure S4: IR-Spectrum of 4-arm CTA. The IR shows no residual alcohol at 3000 cm^{-1} and an ester bond at 1736 cm^{-1} for the 4-arm CTA. The educt pentaerythritol was measured as a control.

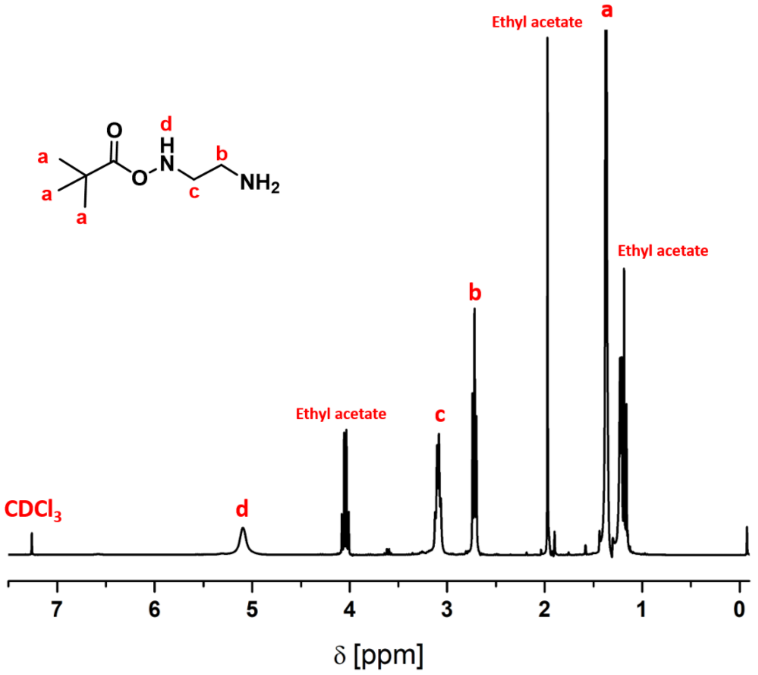

Figure S5: ^{1}H-NMR Spectra of N-t-butoxycarbonyl-1,2-diaminoethane (Solvent $CDCl_3$, 300 MHz).

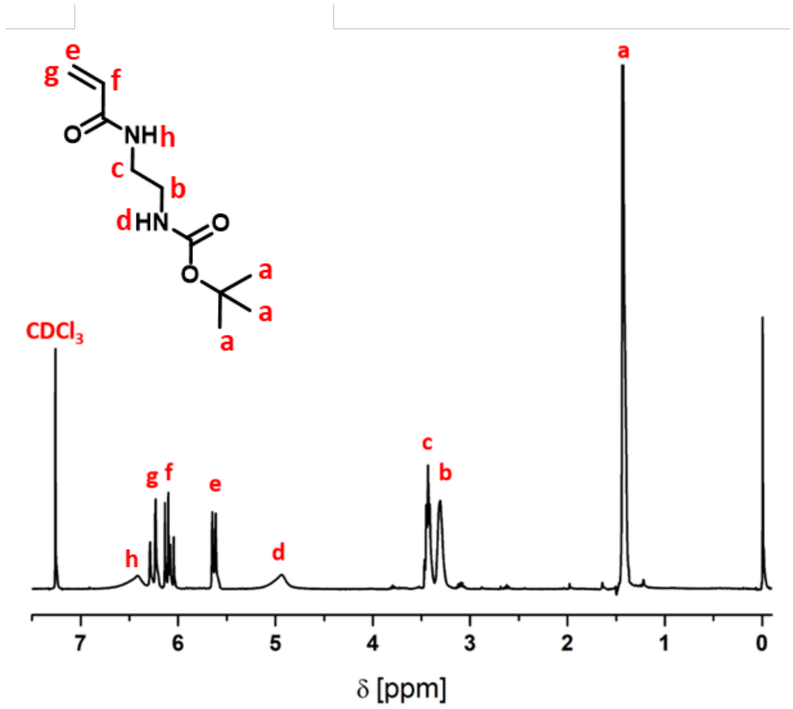

Figure S6: ^{1}H-NMR Spectra of N-t-butoxycarbonyl-N'-acryloyl-1,2-diaminoethane (Solvent $CDCl_3$, 300 MHz).

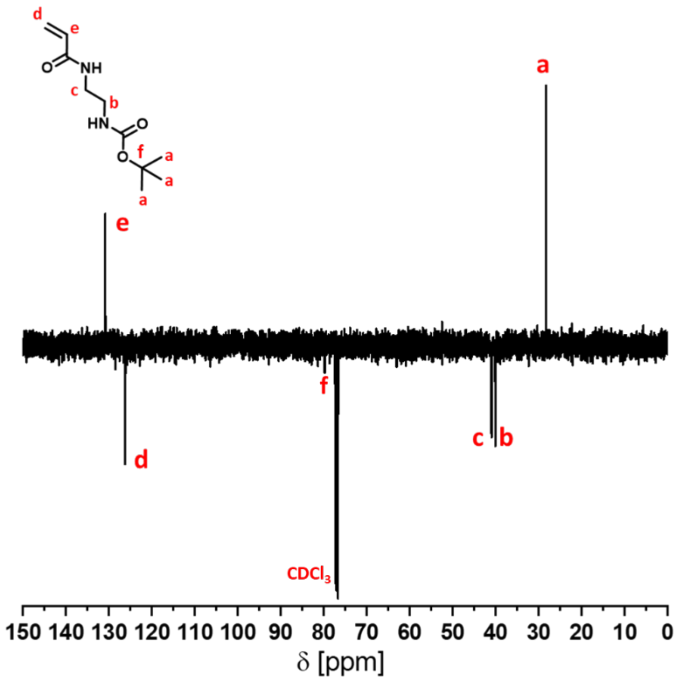

Figure S7: APT ^{13}C-NMR Spectra of N-t-butoxycarbonyl-N'-acryloyl-1,2-diaminoethane (Solvent $CDCl_3$, 400 MHz).

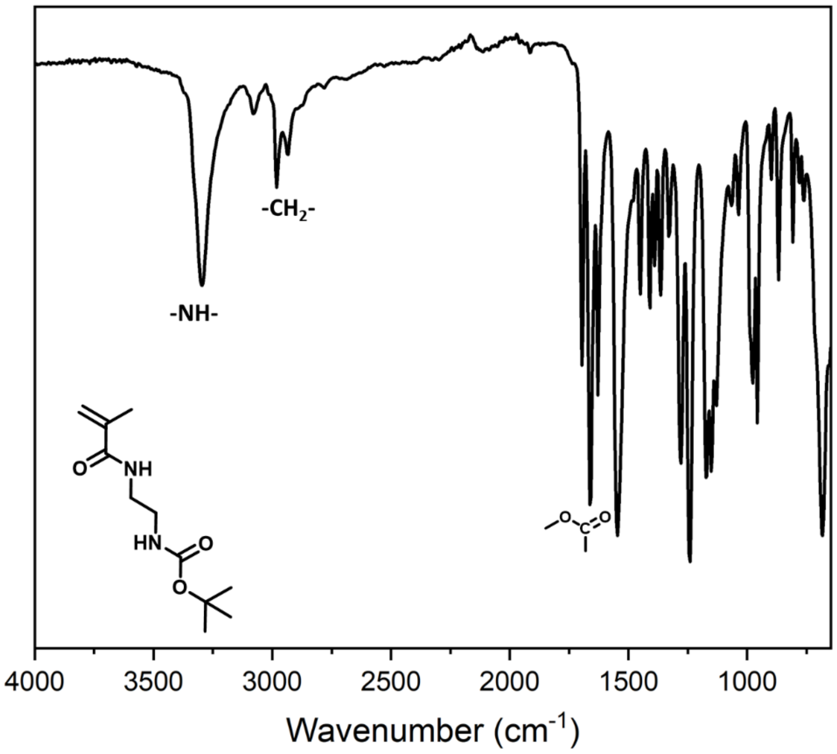

Figure S8: IR-Spectrum of N-t-butoxycarbonyl-N'-acryloyl-1,2-diaminoethane

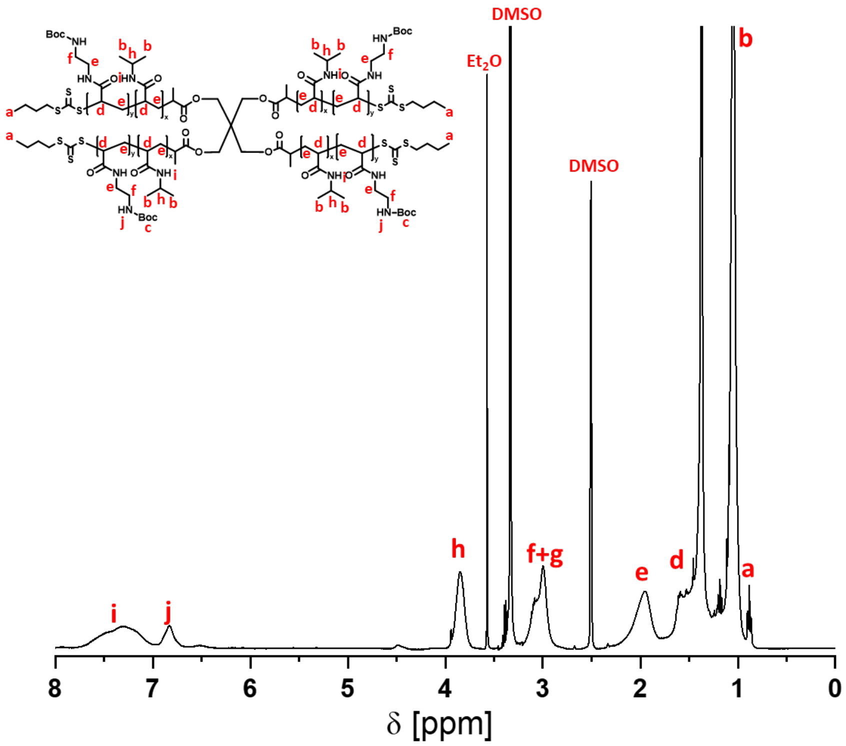

Figure S9: ^{1}H-NMR Spectra of 4-arm star copolymer **S-SP50** (Solvent DMSO-d_6, 400 MHz).

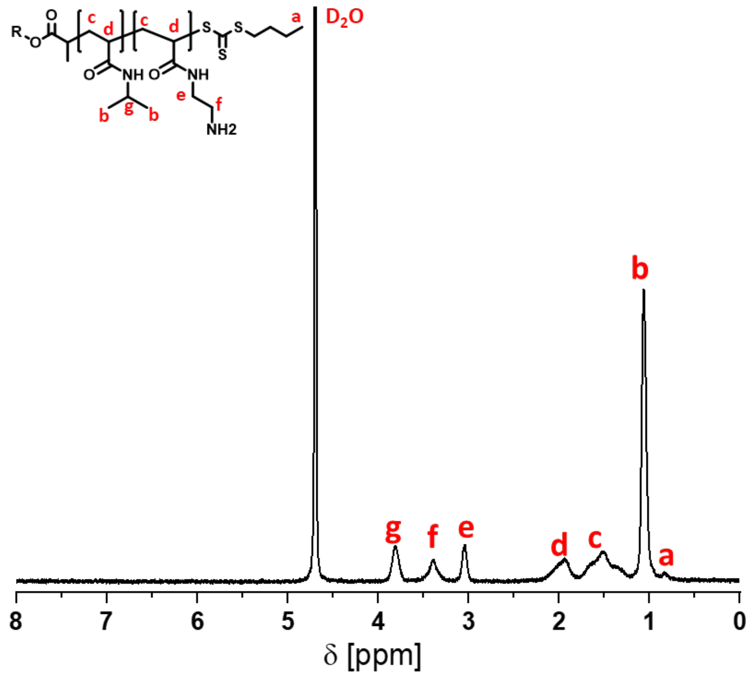

Figure S10: ^{1}H-NMR Spectra of 4-arm star copolymer **S-SP50** deprotected (Solvent D_2O, 400 MHz).

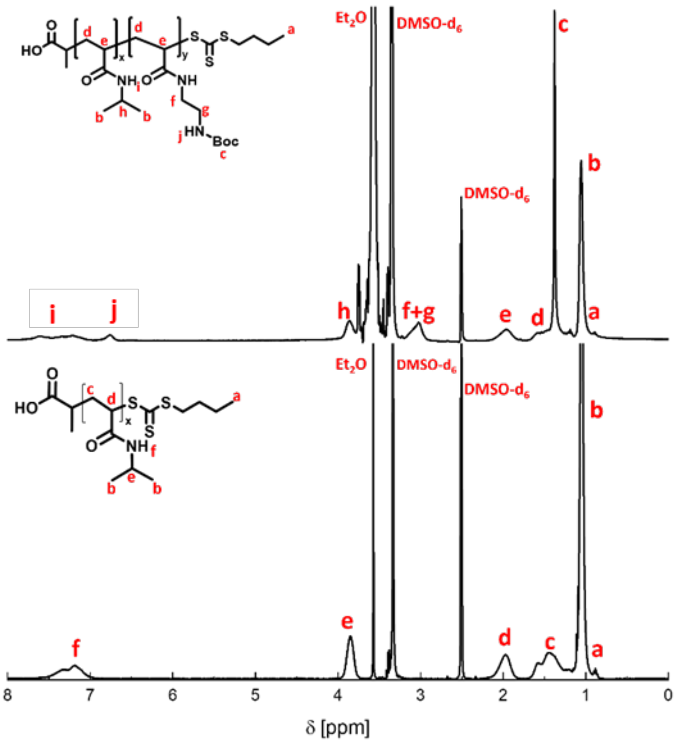

Figure S11: ^{1}H-NMR Spectra of 1st block and chain extension of linear copolymer **D-LP100** (Solvent DMSO-d$_6$, 400 MHz).

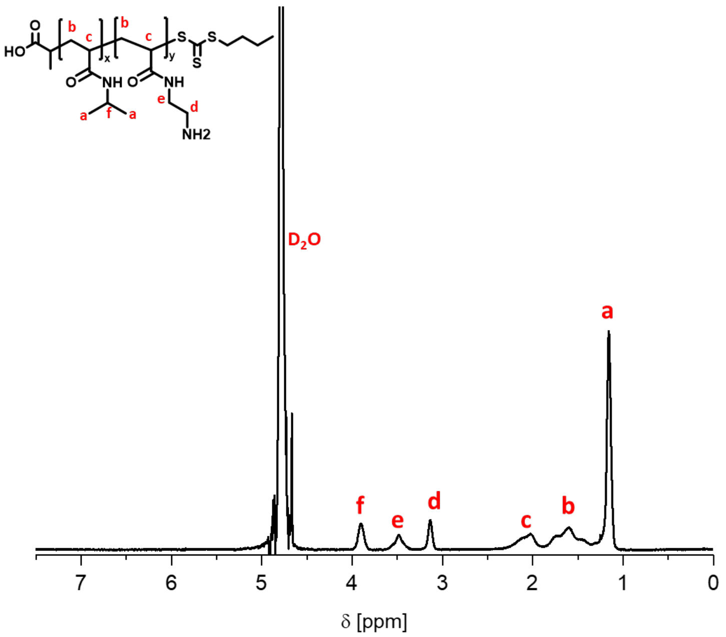

Figure S12: ^{1}H-NMR Spectra of deprotected linear polymer **S-LP100** (Solvent D$_2$O, 400 MHz).

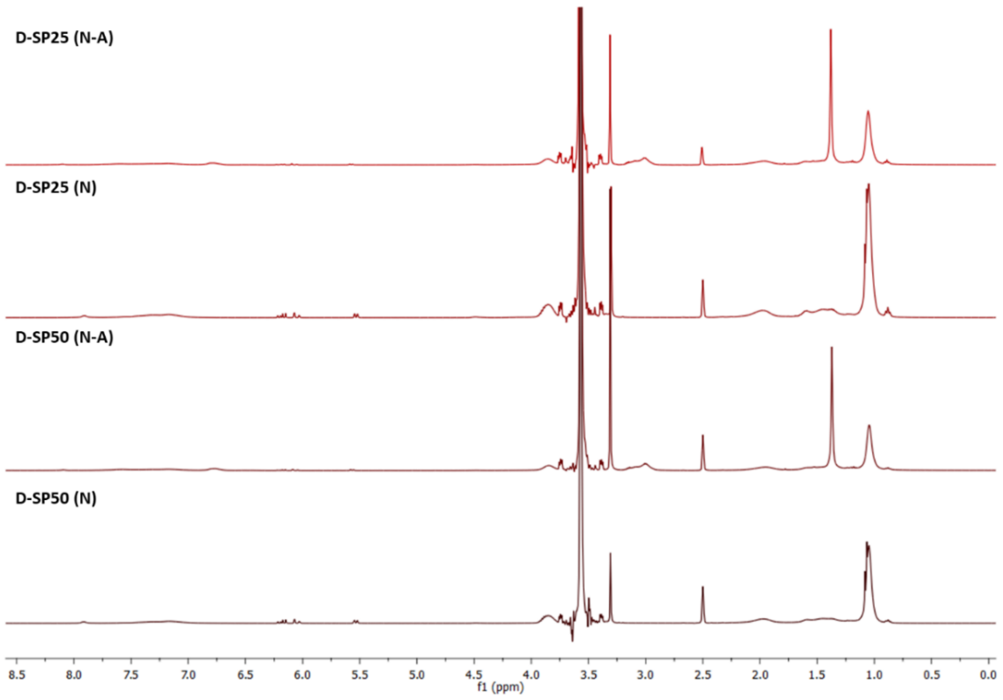

Figure S13: ^{1}H-NMR Spectra in DMSO-d$_6$ of Diblock Star Copolymer Chain extensions for D-SP25 (N-A) and D-SP50 (N-A).

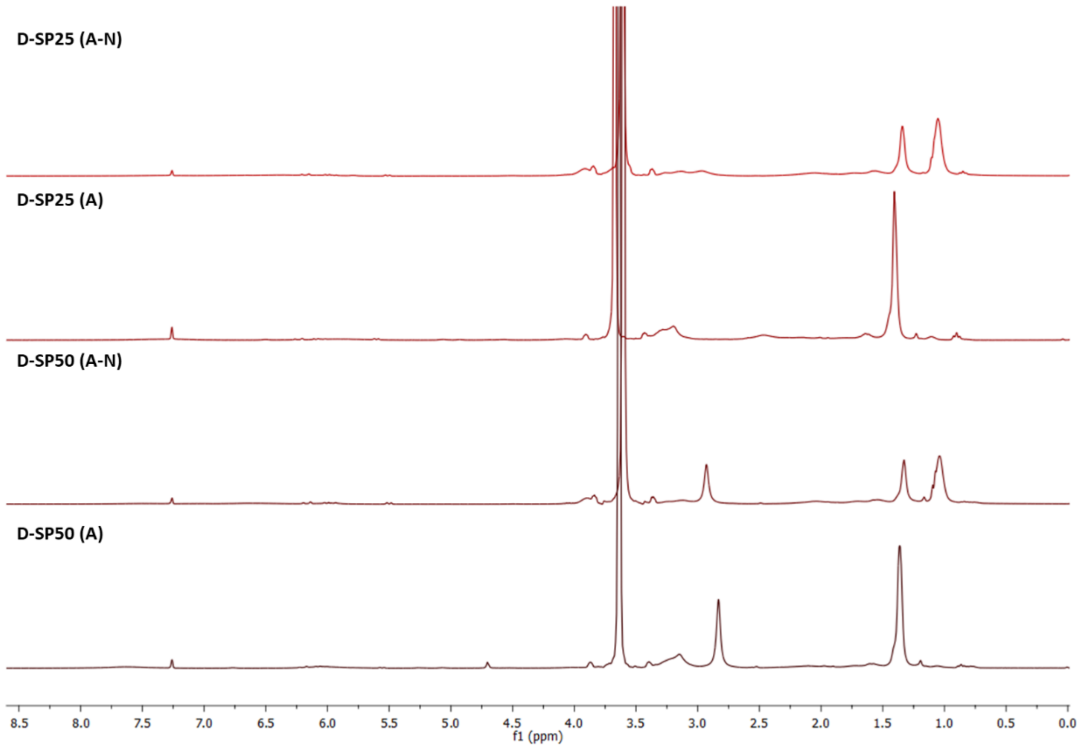

Figure S14: ^{1}H-NMR Spectra in DMSO-d$_6$ of Diblock Star Copolymer Chain extensions for D-SP25 (A-N) and D-SP50 (A-N).

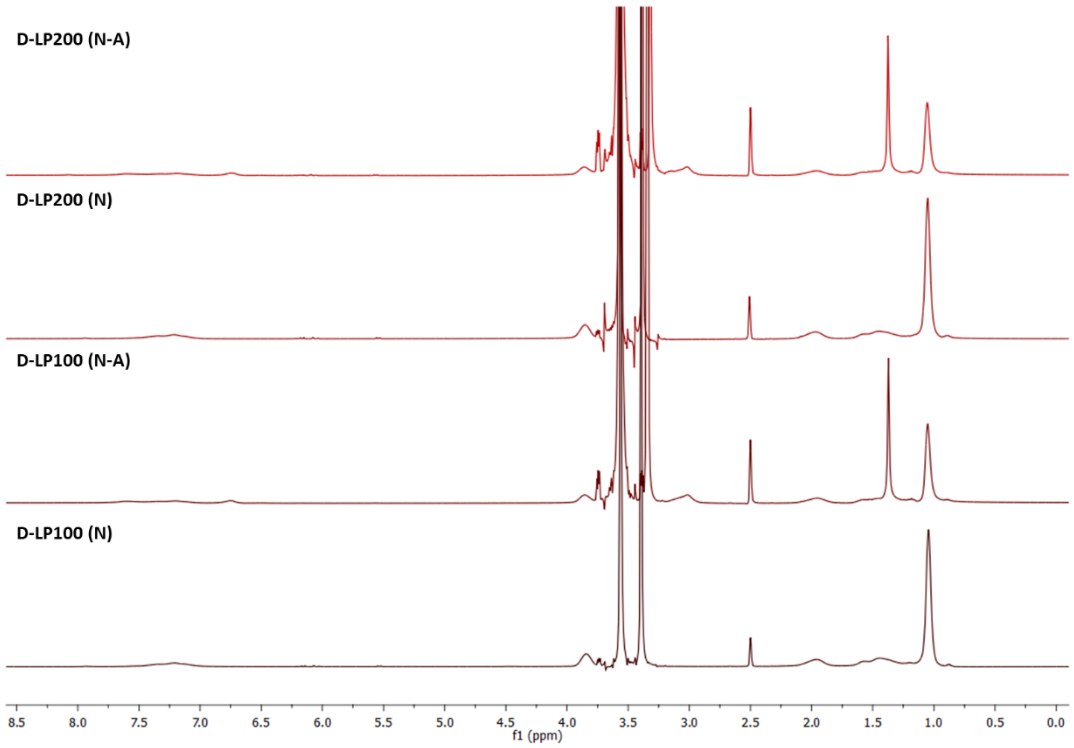

Figure S15: ^{1}H-NMR Spectra in DMSO-d_6 of Diblock Linear Copolymer Chain extensions for D-LP100 and D-LP200.

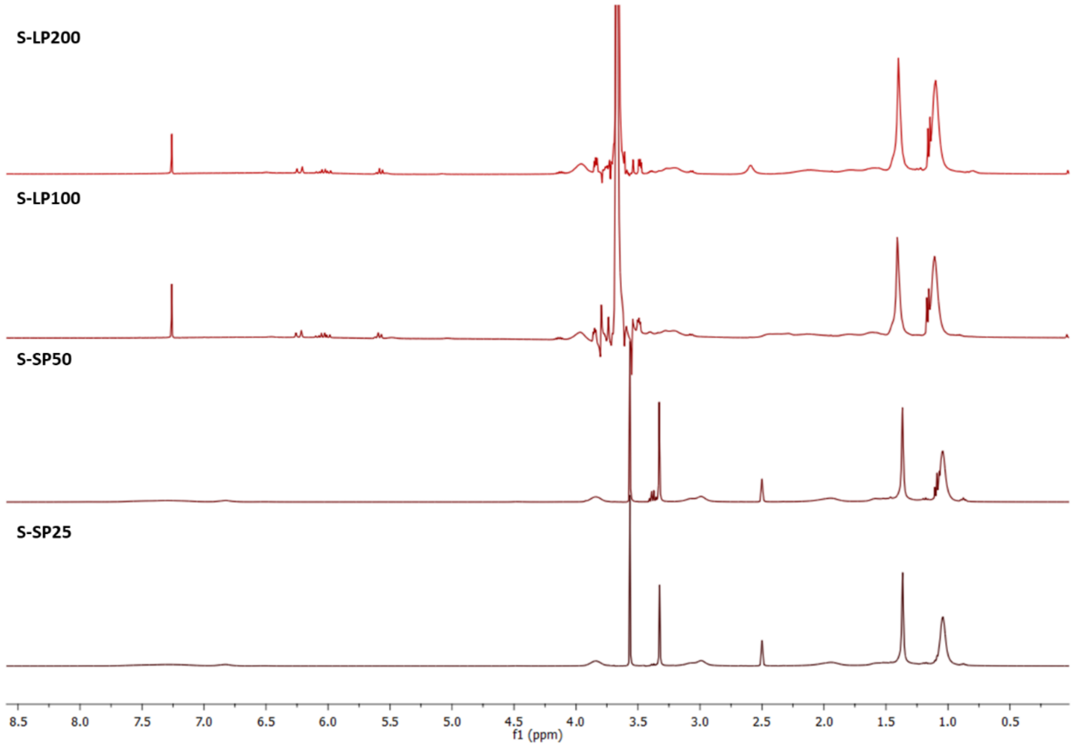

Figure S16: ^{1}H-NMR Spectra of Statistical Star Copolymers S-SP25 and S-SP50 (in DMSO-d_6) and Statistical Linear Polymers S-LP100 and S-LP200 (in $CDCl_3$).

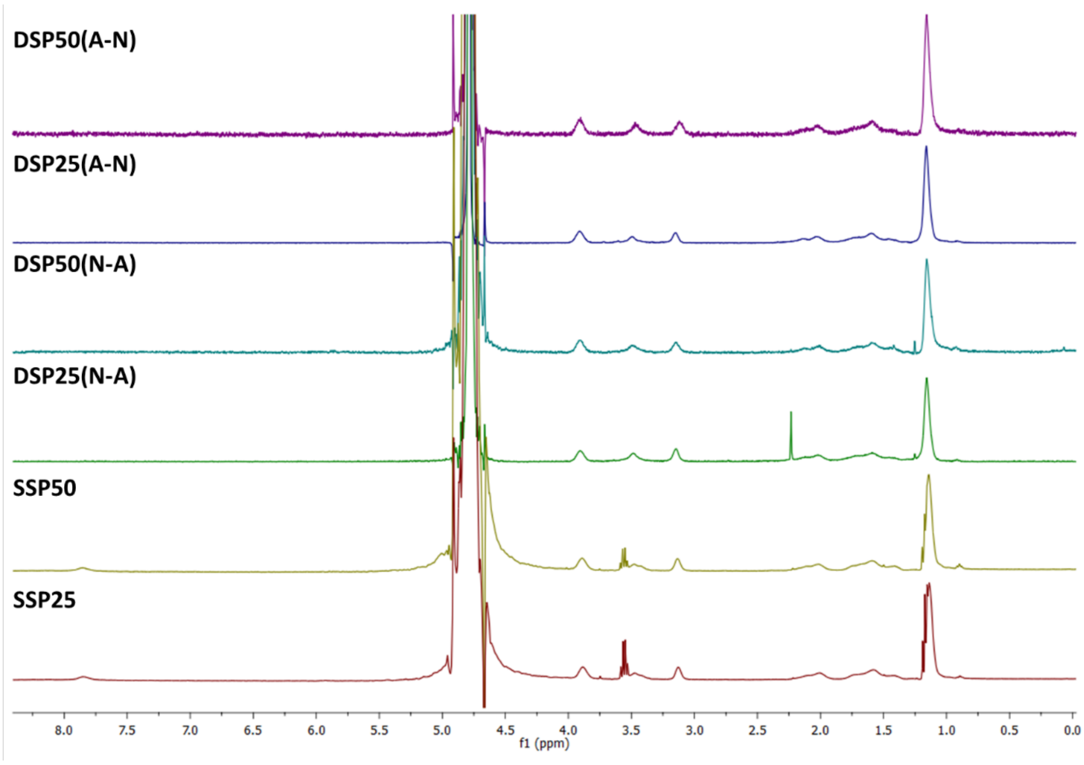

Figure S17: ^{1}H-NMR Spectra of deprotected Star Copolymers (in D_2O).

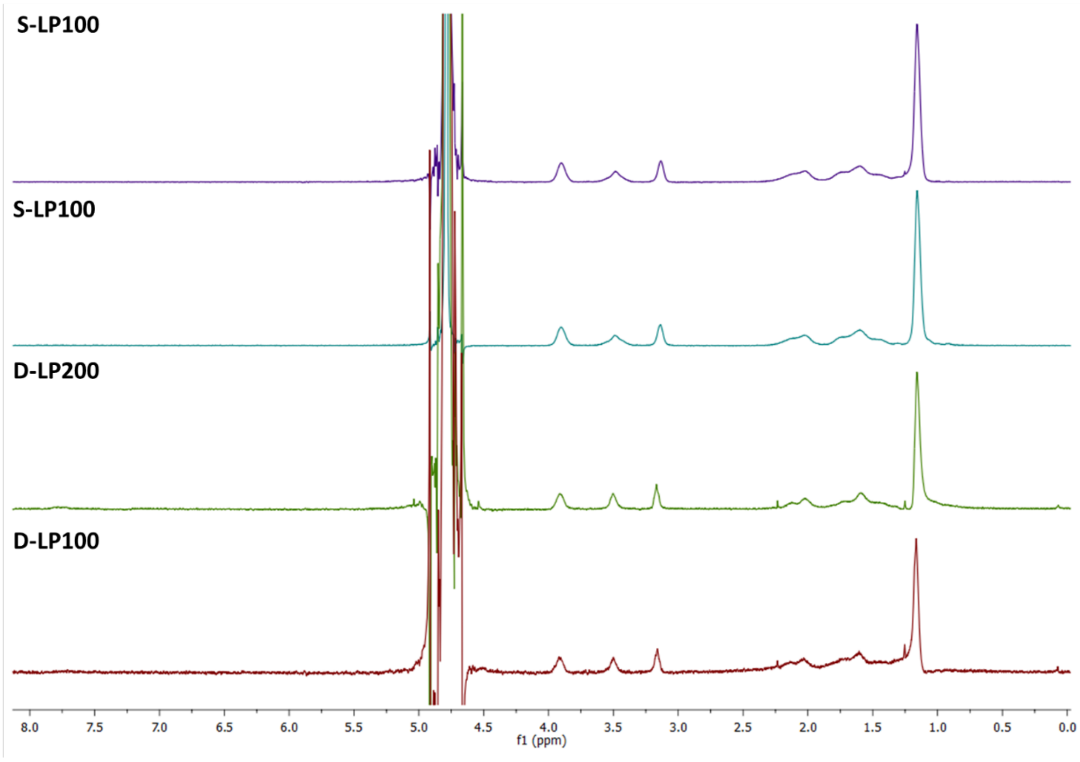

Figure S18: ^{1}H-NMR Spectra of deprotected Linear Copolymers (in D_2O)

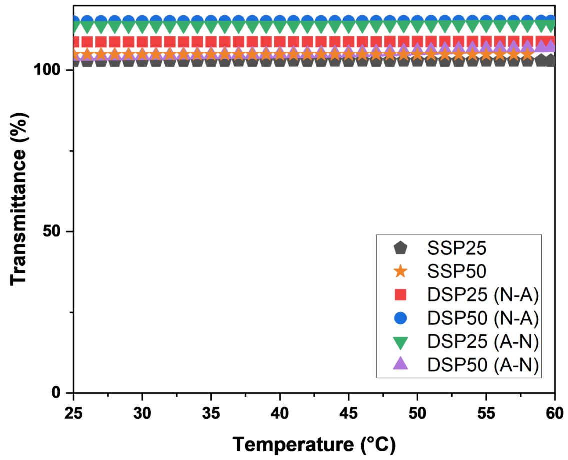

Figure S19: Turbidity measurements for star copolymers, UV-Vis transmittance at 633 nm (2 cycles, 1 °C/min from 25 °C – 60°C).

GPC measurements

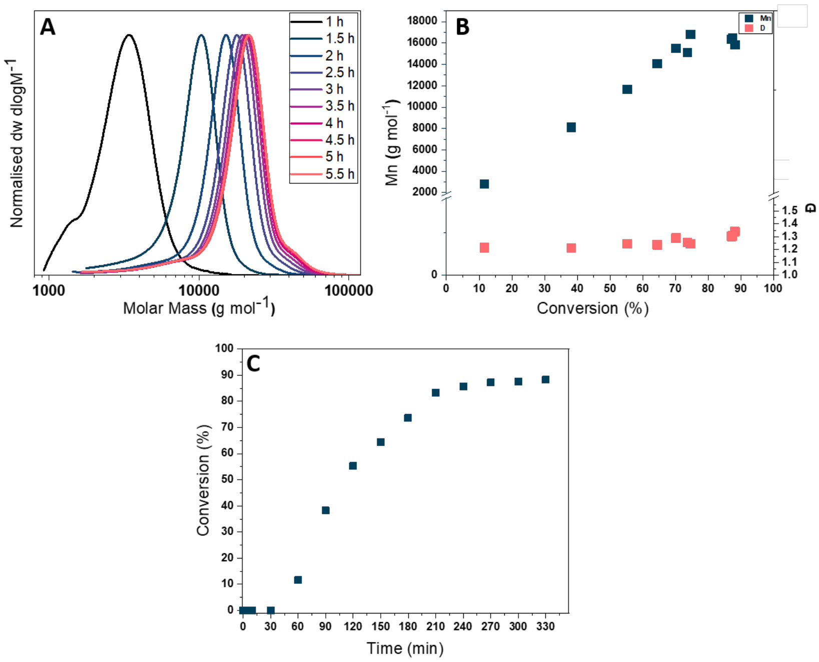

Figure S20: A: SEC measurements of kinetic samples of Homopolymerisation with NIPAM (Eluent: DMF; Calibration PMMA); **B:** Number average molecular weight vs. conversion vs. dispersity plot of kinetic reaction; **C:** Conversion vs. time plot.

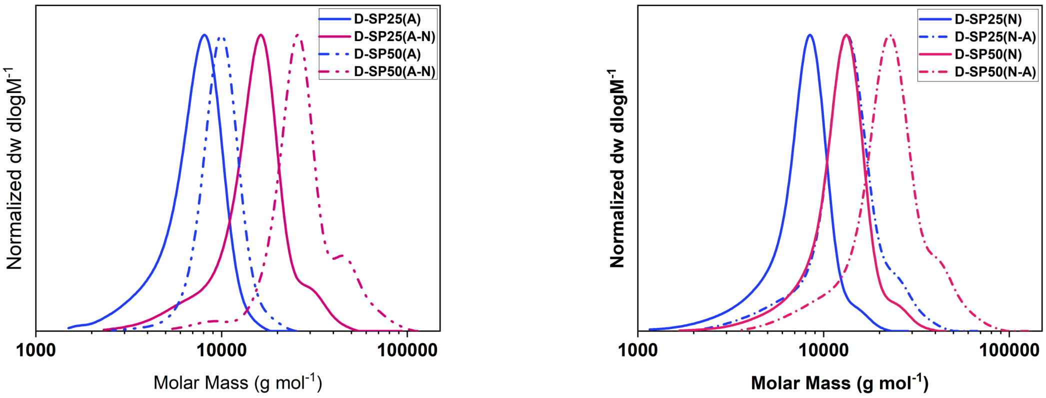

Figure S21: SEC traces of chain extensions of star diblock copolymers D-SP25 (N-A)/(A-N) and D-SP50 (N/A) (A-N) (DMF GPC, PMMA standard).

Biological Assays Raw Data

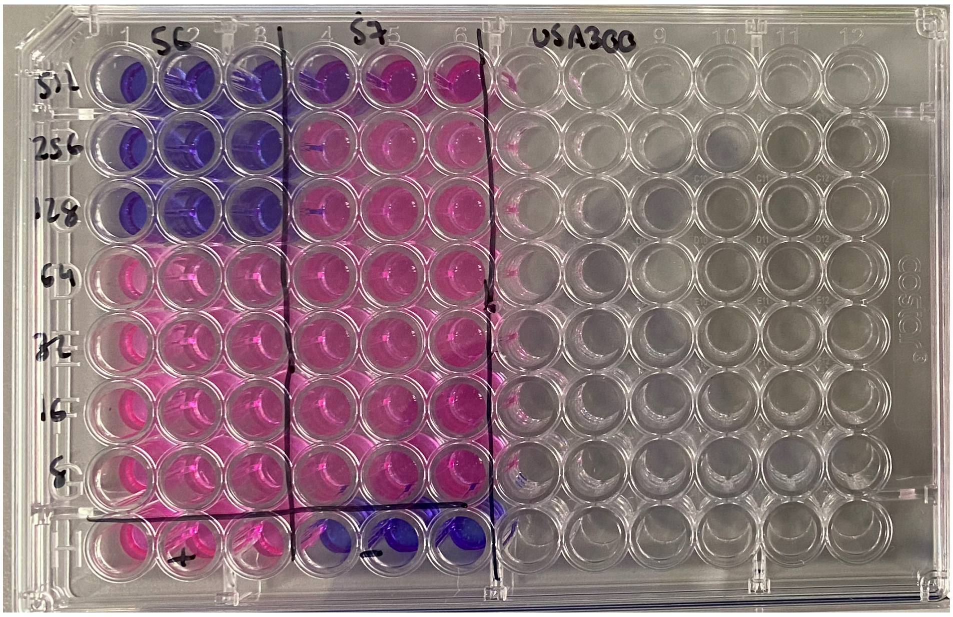

Figure S22: Resazurin Assay for the MIC determination of D-SP25 (N-A) (57) and D-SP50 (N-A) (56) against *S. aureus* USA300 (MIC values determined were 128 µg mL^{-1} for D-SP50 (N-A) and >512 µg mL^{-1} for D-SP25). As positive control (+) S. *aureus* USA300 untreated and as negative control (-) caMHB without bacterial inoculum were used.

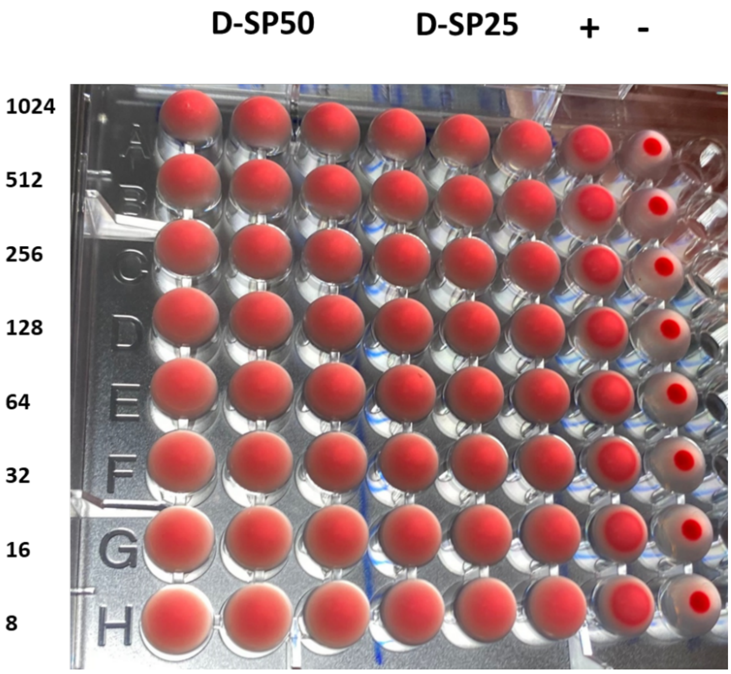

Figure S23: Hemagglutination Assay for D-SP25 (N-A) and D-SP50 (N-A). Concanavalin A was used as the positive control (+) and PBS was used as the negative control (-).

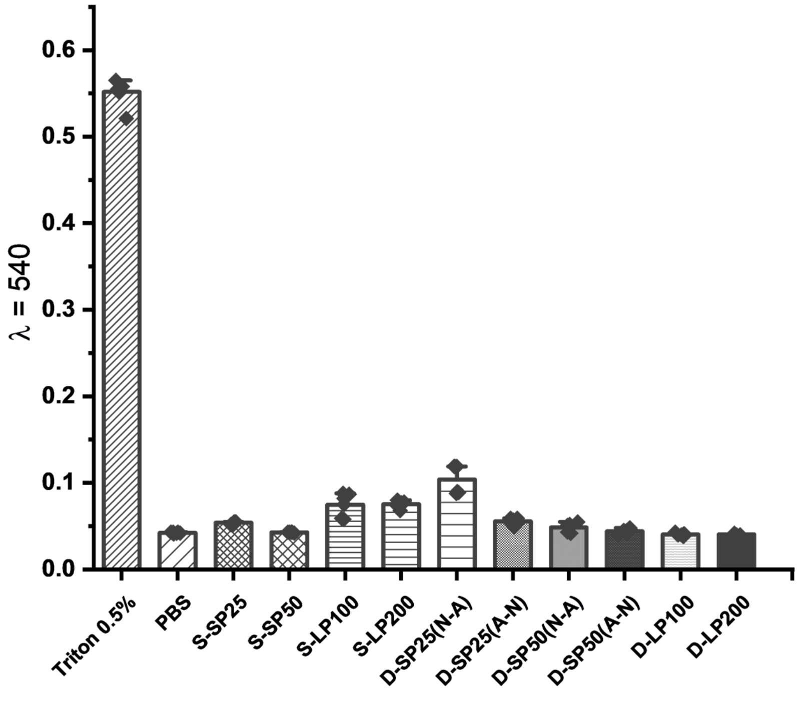

Figure S24: Hemolysis assay absorbance values (sheep RBC in the presence of 1.024 mg/mL of polymers). Triton 0.5% was used as the positive control and PBS was used as the negative control.

Light Microscopy and Scanning Electron Microscopy Images

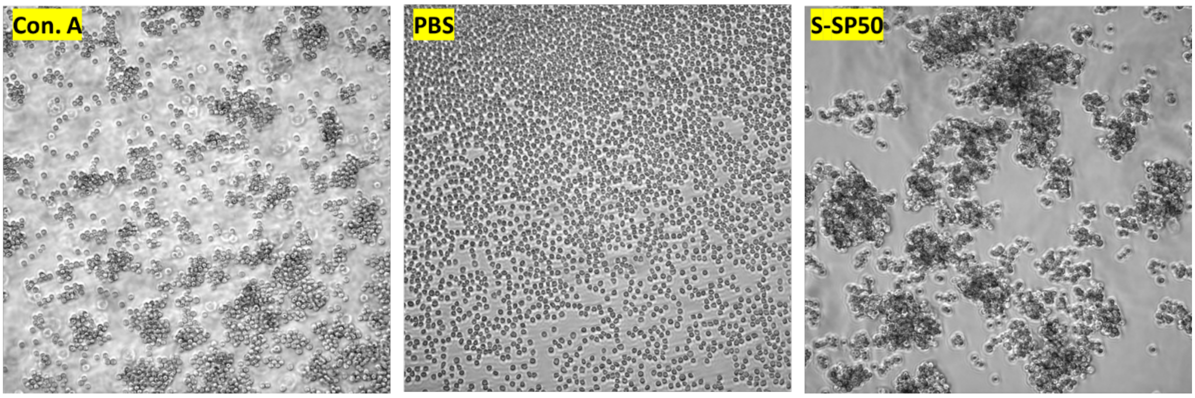

Figure S25: Light Microscope Images of Hemagglutination assay (left to right: Positive control (RBC with Concanavalin A), negative control (RBC with PBS), Star copolymer S-SP50 at 8 µg mL^{-1}).

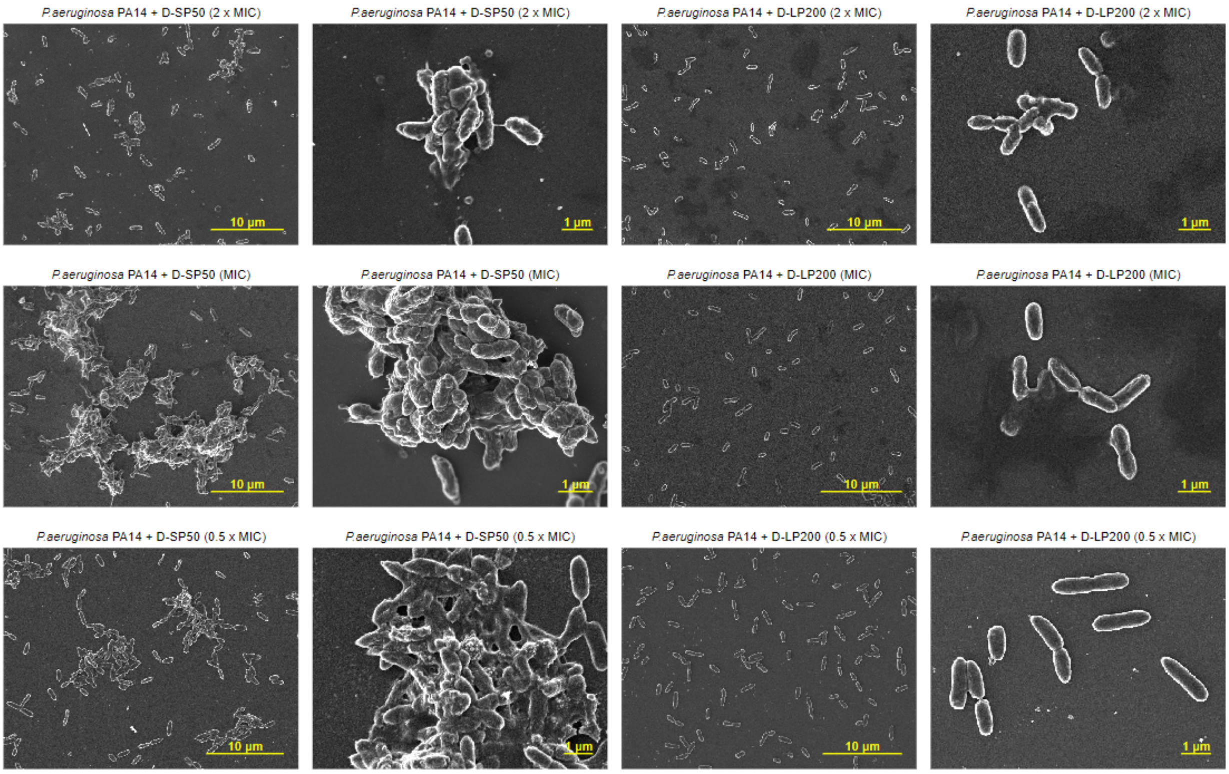

Figure S26: Scanning electron micrographs of *P. aeruginosa* treated with D-SP50 and D-LP200 at 0.5xMIC, MIC and 2xMIC for 1h.

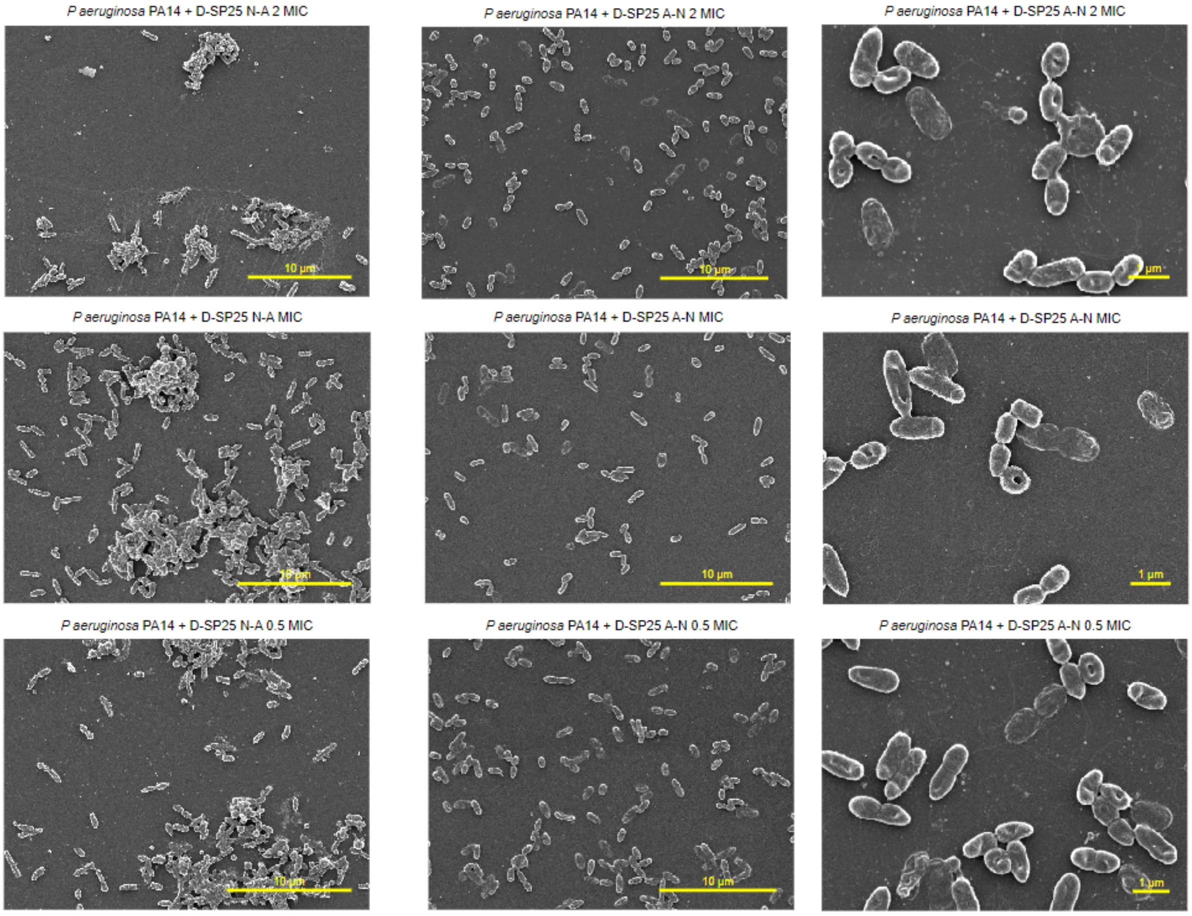

Figure S27: Scanning electron micrographs of *P. aeruginosa* treated with D-SP25(N-A) and D-SP25(A-N) at 0.5xMIC, MIC and 2xMIC for 1h.

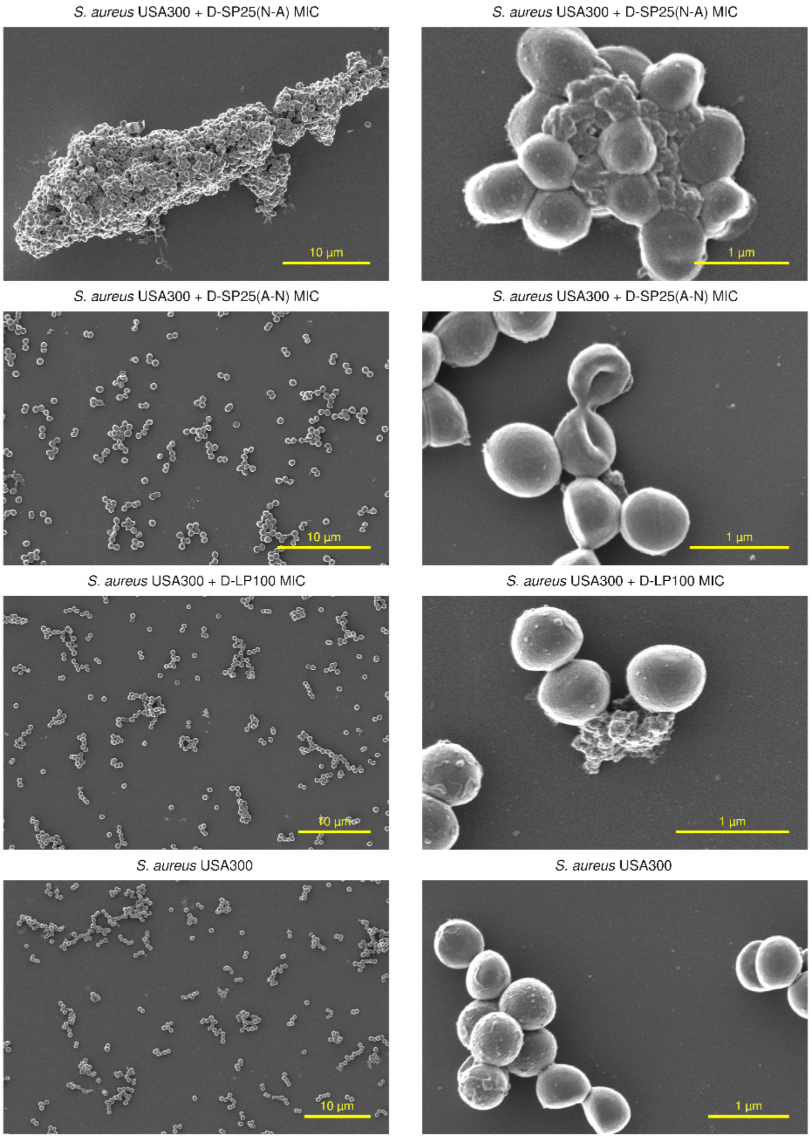

Figure S28: Scanning electron micrographs of *S. aureus* treated with D-SP25(N-A), D-SP25(N-A) and D-LP100 at MIC for 1h. D-SP25(A-N) and D-SP25(N-A) did not show activity within concentration range tested, highest concentration of polymer was used (512 µg mL^{-1}).

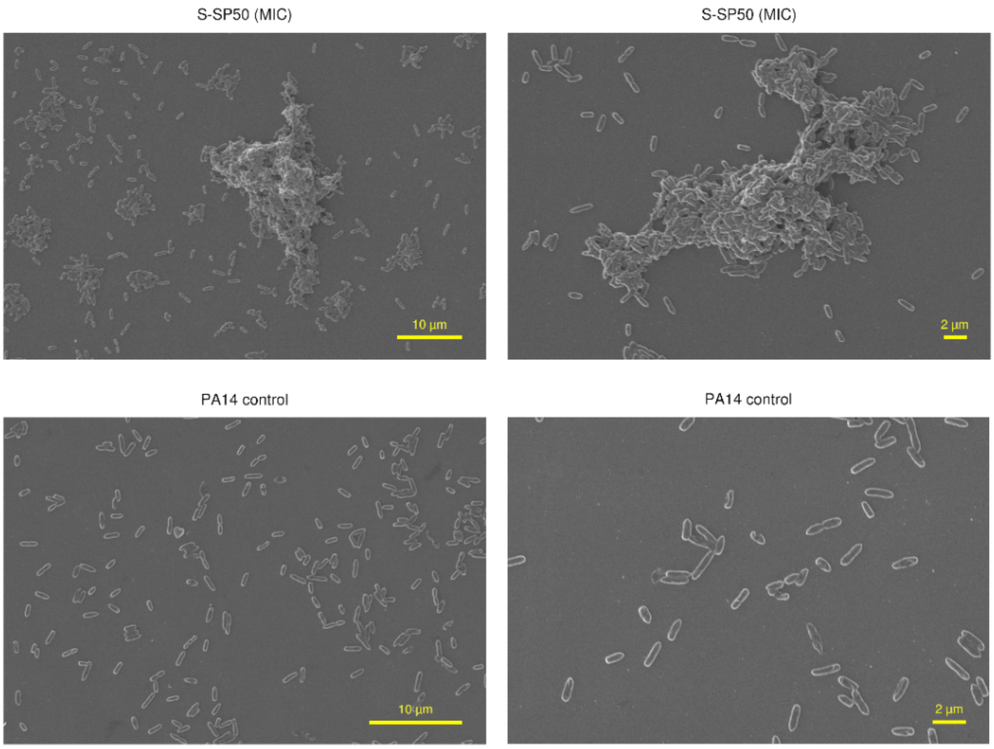

Figure S29 Scanning electron micrographs of *P. aeruginosa* treated with S-SP50 at MIC and with caMHB (untreated control) for 1h.

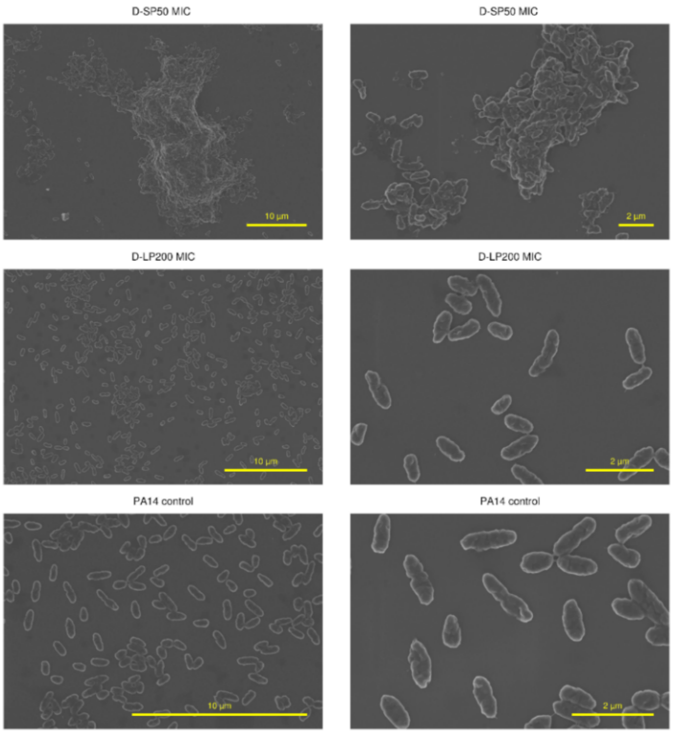

Figure S30: Scanning electron micrographs of boiled P. aeruginosa PA14 incubated with D-SP50 and D-LP200 or caMHB (untreated control) at MIC concentrations for 1h.
